# Soybean transporter AAT *_Rhg1_* abundance increases along the nematode migration path and impacts vesiculation and ROS

**DOI:** 10.1093/plphys/kiad098

**Published:** 2023-02-20

**Authors:** Shaojie Han, John M Smith, Yulin Du, Andrew F Bent

**Affiliations:** Department of Plant Pathology, University of Wisconsin—Madison, Madison, WI 53705, USA; Ministry of Agriculture Key Laboratory of Molecular Biology of Crop Pathogens and Insect Pests, Institute of Biotechnology, College of Agriculture and Biotechnology, Zhejiang University, Hangzhou 310058, China; Zhejiang Lab, Hangzhou 311121, China; Department of Plant Pathology, University of Wisconsin—Madison, Madison, WI 53705, USA; Department of Plant Pathology, University of Wisconsin—Madison, Madison, WI 53705, USA; Department of Plant Pathology, University of Wisconsin—Madison, Madison, WI 53705, USA

## Abstract

*Rhg1* (Resistance to *Heterodera glycines* 1) mediates soybean (*Glycine max*) resistance to soybean cyst nematode (SCN; *H. glycines*). *Rhg1* is a 4-gene, ∼30-kb block that exhibits copy number variation, and the common PI 88788-type *rhg1-b* haplotype carries 9 to 10 tandem *Rhg1* repeats. *Glyma.18G022400* (*Rhg1-GmAAT*), 1 of 3 resistance-conferring genes at the complex *Rhg1* locus, encodes the putative amino acid transporter AAT_Rhg1_ whose mode of action is largely unknown. We discovered that AAT_Rhg1_ protein abundance increases 7- to 15-fold throughout root cells along the migration path of SCN. These root cells develop an increased abundance of vesicles and large vesicle-like bodies (VLB) as well as multivesicular and paramural bodies. AAT_Rhg1_ protein is often present in these structures. AAT_Rhg1_ abundance remained low in syncytia (plant cells reprogrammed by SCN for feeding), unlike the *Rhg1* α-SNAP protein, whose abundance has previously been shown to increase in syncytia. In *Nicotiana benthamiana*, if soybean AAT_Rhg1_ was present, oxidative stress promoted the formation of large VLB, many of which contained AAT_Rhg1_. AAT_Rhg1_ interacted with the soybean NADPH oxidase GmRBOHG, the ortholog of *Arabidopsis thaliana* RBOHD previously found to exhibit upregulated expression upon SCN infection. AAT_Rhg1_ stimulated reactive oxygen species (ROS) generation when AAT_Rhg1_ and GmRBOHG were co-expressed. These findings suggest that AAT_Rhg1_ contributes to SCN resistance along the migration path as SCN invades the plant and does so, at least in part, by increasing ROS production. In light of previous findings about α-SNAP_Rhg1_, this study also shows that different *Rhg1* resistance proteins function via at least 2 spatially and temporally separate modes of action.

## Teaser

AAT_Rhg1_ (resistance-conferring putative transporter encoded by soybean *Rhg1*) raises vesiculation and ROS generation and becomes abundant along the soybean cyst nematode migration path in roots.

## Introduction

*Rhg1* (Resistance to *H. glycines* 1) of soybean (*Glycine max*) is a complex genetic locus that encodes novel mechanisms of disease resistance (Cook et al. 2012; Bayless et al. 2016; Mitchum 2016). *Rhg1* is a central tool used for the control of soybean cyst nematode (SCN, *H. glycines*), the most economically damaging pathogen of US soybeans (Niblack et al. 2006; Jones et al. 2013; Mitchum 2016; Allen et al. 2017). The mechanisms of *Rhg1* activity remain only partially understood.

During pathogenesis, recently hatched J2 SCN migrate toward soybean root exudates and then penetrate soybean roots above the root cap in the zone of elongating cells (Endo 1992). They then migrate intracellularly through root cortical cells, secreting bioactive effectors through their stylet to degrade cell walls and manipulate plant defense responses (Qin et al. 2004; Gheysen and Mitchum 2011; Mitchum et al. 2013; Sato et al. 2019). The protrusible stylet is also used directly to disrupt plant cell walls prior to cell penetration. When it reaches a suitable site adjacent to the vascular cylinder the nematode selects an individual endodermis or endodermis-adjacent cortical cell upon which to feed. Over subsequent days the cell walls are partially dissolved between an increasingly large cluster of dozens of adjacent root cells that also lose their large central vacuoles and become metabolically hyperactive, forming a multinucleate syncytium from multiple cytoplasmically merged cells (Jones 1981; Fenoll et al. 1997; Kyndt et al. 2013). After 3 to 4 wk of deriving nutrients from the host through a syncytium, the life cycle of a fertilized female SCN is completed by forming an egg-filled and durable cyst. Despite a number of previous studies (e.g. Mahalingam and Skorupska 1996; Hermsmeier et al. 1998; Escobar et al. 2011; Kandoth et al. 2011), there is particularly incomplete knowledge regarding how plants respond during the early stages of infection as SCN penetrate and migrate through relatively new root tissues.

The structure of the complex *Rhg1* locus has been characterized across a wide array of soybean germplasm (Cook et al. 2012, 2014; Lee et al. 2015; Patil et al. 2019). Increased copy number of a 4-gene block is a hallmark of resistance-conferring *Rhg1* haplotypes (Cook et al. 2012). “Peking”-type (*rhg1-a*) haplotypes typically carry 3 copies while “PI 88788”-type (*rhg1-b*) haplotypes often carry 9 or 10 copies of the ∼30 kb *Rhg1* 4-gene block (Cook et al. 2012, 2014; Lee et al. 2015). *rhg1-a* generally must be combined with an appropriate allele of the unlinked *Rhg4* and/or *Rhg2* loci to achieve sufficient SCN resistance (Brucker et al. 2005; Liu et al. 2012; Mitchum 2016; Basnet et al. 2022). Until recent decades most soybean accessions carried single-copy “Williams 82”-type *Rhg1_WT_* loci and were SCN-susceptible (Niblack et al. 2008; Lee et al. 2015; Bayless et al. 2019). In the current US market, approximately 95% of SCN-resistant soybean rely on the PI88788-derived *rhg1-b* haplotype (Niblack et al. 2008; Tylka and Mullaney 2015; Rincker et al. 2017)

Gene silencing and gene overexpression approaches have demonstrated individual contributions to SCN resistance by 3 of the 4 genes within the multicopy *Rhg1* segment (Cook et al. 2012; Liu et al. 2017; Butler et al. 2019; Dong and Hudson 2021). The resistance-contributing *Rhg1* genes include the subject of the present study, *Rhg1-GmAAT* (*Glyma.18G022400*, formerly named *Glyma18g02580*), which encodes a putative amino acid transporter hereafter referred to as *AAT_Rhg1_* (Cook et al. 2012). Contributions to SCN resistance have also been documented for the adjacent *Rhg1* genes *Glyma.18G022500* (formerly named *Glyma18g02590*), encoding a predicted α-SNAP (alpha-soluble NSF [*N*-ethylmaleimide-sensitive factor] attachment protein), and *Glyma.18G022700* (formerly named *Glyma18g02610*), encoding a protein with a WI12 wound-inducible protein domain (Cook et al. 2012; Liu et al. 2017; Butler et al. 2019; Dong and Hudson 2021). The contributions of AAT_Rhg1_ and α-SNAP_Rhg1_ (also known as GmSNAP18) to restriction of SCN progression past the J2 stage in transgenic soybean roots were quantitatively similar, while that of WI12_Rhg1_ was possibly greater (Fig. 1B of Cook et al. 2012). Several recent studies on the *Rhg1*-encoded α-SNAP proteins have revealed their elevated abundance in syncytia and their cytotoxicity, which apparently poisons syncytium cells during the otherwise biotrophic plant-nematode interaction (Cook et al. 2012; Matsye et al. 2012; Cook et al. 2014; Bayless et al. 2016; Lakhssassi et al. 2017; Liu et al. 2017; Bayless et al. 2018, 2019).

Until 2 recent publications, the products of the other 2 *Rhg1* genes that contribute to SCN resistance, *Glyma.18G022400* (AAT_Rhg1_) and *Glyma.18G022700* (WI12_Rhg1_), had been much less well characterized (Guo et al. 2019; Dong and Hudson 2021). There are no predicted amino acid polymorphisms in the products of those genes between SCN-susceptible *Rhg1_WT_* and the resistance-conferring low-copy *rhg1-a* and high-copy *rhg1-b* haplotypes. However, the higher copy numbers of those *Rhg1* genes result in constitutive elevation of their transcript abundance in SCN-resistant plants (Cook et al. 2014), and locus copy number has been shown to correlate with the strength of SCN resistance conferred by various *rhg1-a* and *rhg1-b* haplotypes (Cook et al. 2014; Lee et al. 2016; Yu et al. 2016; Patil et al. 2019). mRNA encoding AAT_Rhg1_ was reported to be more abundant upon SCN infection (Kandoth et al. 2011; Matsye et al. 2011). A higher accumulation of resistance-associated α-SNAP_Rhg1_ in syncytia was reported to play a key role in poisoning the nematode during development. However, the accumulation pattern of AAT_Rhg1_ was not known.

The amino acids transported by AAT_Rhg1_, if any, are not known. Similarly, very little is known about *AT3G56200*/AtAVT6C, the *Arabidopsis thaliana* ortholog of soybean AAT_Rhg1_. The family is predicted to export neutral amino acids including aspartate and glutamate, and a recent study of the related protein AtAVT6D reported plasma membrane localization in *Nicotiana benthamiana* and aspartate transport in *Xenopus* (Dhatterwal et al. 2022). AAT_Rhg1_ retains the sequence hallmarks of a bona fide amino acid transporter but, in unpublished work done in the laboratories of our collaborators, AAT_Rhg1_ has been recalcitrant in yeast and *Xenopus* oocyte experiments attempting to document the transport of amino acids (A. Reinders, J.M. Ward, B.E. Broeckling, and D.R. Bush, unpublished data). A recent publication showed that overexpression of *Rhg1-GmAAT* in soybean can increase tolerance of toxic levels of exogenously supplied glutamate, and presented additional indirect evidence showing that AAT_Rhg1_ impacts glutamate abundance and transport (Guo et al. 2019). Endogenous jasmonic acid (JA) levels and JA pathway genes were also upregulated in *Rhg1-GmAAT* overexpression soybean lines (Guo et al. 2019). However, evidence is still lacking regarding mechanistic roles of AAT_Rhg1_ in SCN-soybean interactions.

Reactive oxygen species (ROS) are commonly produced during plant–pathogen interactions and act up- and downstream of various signaling pathways (Apel and Hirt 2004; Camejo et al. 2016; Waszczak et al. 2018). Mechanisms that control ROS production during infections, and the impacts of ROS on infection outcomes, are diverse and continue to be discovered. ROS generated during pattern-triggered immunity (PTI) act as important defense signal transduction molecules (Macho and Zipfel 2014). ROS generated from extracellular and/or intracellular sources can accumulate to more toxic levels during the hypersensitive response associated with effector-triggered immunity (ETI) (Zurbriggen et al. 2010). Plant ROS production can also contribute to disease susceptibility, for example, in soybean interactions with necrotrophic *Sclerotinia sclerotiorum* (Ranjan et al. 2018). Moderate levels of ROS can help limit the extent of cell death (Torres et al. 2005). This appears to be the case during colonization of Arabidopsis by beet cyst nematodes, for which ROS responses were shown to help limit plant cell death and enhance nematode growth (Siddique et al. 2014; Chopra et al. 2021). In other cases, ROS have been shown to contribute to plant defense against plant parasitic nematodes (Simonetti et al. 2010; Kandoth et al. 2011; Kong et al. 2015; Pant et al. 2015; Teixeira et al. 2016; Habash et al. 2017; Labudda et al. 2018; Lee et al. 2018; Mei et al. 2018; Zhou et al. 2018; Yang et al. 2019a, 2019b; Chen et al. 2020; Hawamda et al. 2020; Labudda et al. 2020).

Plasma membrane-localized NADPH oxidases, encoded by respiratory burst oxidase homolog genes (*RBOH*), are key enzymes for pathogenesis-associated ROS generation (Averyanov 2009). In soybean, the 17 *GmRBOH* genes were recently characterized by 2 separate groups (Ranjan et al. 2018; Liu et al. 2019), including their stress induction patterns. In Arabidopsis, a particular RBOH, RBOHD, plays major roles in mediating ROS production during both PTI and ETI (Kadota et al. 2015). As one of many examples, AtRBOHD interacts with the FLS2 immune receptor complex and is phosphorylated by BIK1 to enhance ROS generation that contributes to stomatal closure defense mechanisms against *Pseudomonas* bacteria (Li et al. 2014). RBOHD orthologs in multiple plant species have been reported to control PTI (e.g. (Simon-Plas et al. 2002; Yoshioka et al. 2003; Trujillo et al. 2006; Kobayashi et al. 2007; Wong et al. 2007; Li et al. 2015; Lee et al. 2020)). RBOHD contributes to defense against root-knot nematodes (Teixeira et al. 2016; Zhou et al. 2018). Soybean *GmRBOHG* (*Glyma.06G162300*) is one of the orthologs of Arabidopsis *RBOHD* (Ranjan et al. 2018; Liu et al. 2019). *GmRBOHG* is the only RBOH mRNA reported to increase more than 2-fold during SCN infection in both susceptible and resistant soybean lines (Wan et al. 2015; Liu et al. 2019), but other aspects of GmRBOHG behavior during SCN infestations have not been characterized.

The present study investigated soybean AAT_Rhg1_. We discovered a unique accumulation of this protein along the SCN root penetration/migration path. Extensive vesiculation occurred in soybean root cortical cells penetrated during SCN migration and AAT_Rhg1_ was often present on those large vesicle-like bodies (VLB). Overexpression of *Rhg1-GmAAT* in *N. benthamiana* also caused AAT_Rhg1_ association with a subset of the extensively present VLB. Further, we found that AAT_Rhg1_ interacts with GmRBOHG, a soybean ortholog of Arabidopsis RBOHD. Simultaneous overexpression of AAT_Rhg1_ and GmRBOHG in *N. benthamiana* elevated ROS production. The elevated abundance of AAT_Rhg1_ along the SCN migration path contrasts with the elevated abundance of *Rhg1*-encoded α-SNAP_Rhg1_ in syncytia. Hence the α-SNAP_Rhg1_ and AAT_Rhg1_ proteins apparently contribute to SCN resistance through temporally, spatially, and biochemically distinct mechanisms.

## Results

### Soybean AAT_Rhg1_ protein abundance is elevated along the migration path of SCN

**Figure 1.**
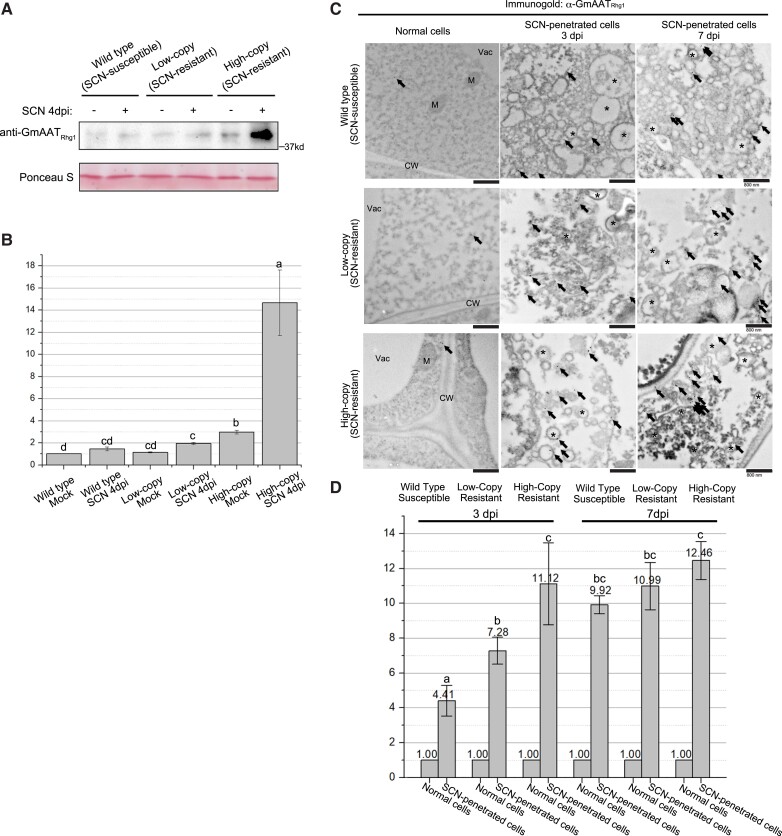
AAT_Rhg1_ protein abundance increase in SCN-penetrated soybean root cells, with AAT_Rhg1_ often localized on vesicles and giant vesicles induced by SCN migration. **A)** Representative immunoblot showing the abundance of AAT_Rhg1_ protein in soybean lines that carry *rhg1-c* (WT), *rhg1-a* (low-copy), or *rhg1-b* (high-copy) haplotypes. Detached roots from the 3 varieties were either infected with an Hg Type 0 population of SCN (SCN +) or mock-inoculated with 0.05% sterile agarose water (SCN −), and harvested at 4 dpi. Samples of SCN-infested root regions from 4 roots per treatment were pooled together for the immunoblot. Ponceau staining of the blotted membrane shown as a check for equivalent loading of total protein. **B)** Densitometry analysis of immunoblot AAT_Rhg1_ protein levels. *N* = 4 for each treatment; data obtained from 2 biological replicates. Band intensity was normalized to the intensity for WT mock treatment within each blot; mean ± SE are shown. Bars with the same letter are not significantly different (ANOVA Tukey analysis performed on non-normalized data, *P* < 0.05). **C)** Representative electron micrographs taken at 15,000 × magnification showing immunogold-labeled AAT_Rhg1_ (solid black particles) on vesicle membranes of SCN-penetrated cells in SCN-infested roots of susceptible (upper panels), *rhg1-a* SCN resistant (low-copy, middle panels), and *rhg1-b* SCN resistant (high-copy, bottom panels) genotypes, at 3 dpi (middle column) and 7 dpi (right column), but not in normal cells (left column) from the same samples. Arrows indicate immunogold particles in each image. Asterisks highlight vesicle clusters in SCN-penetrated cells. CW, cell wall; M, mitochondrion; Vac, vacuole. All scale bars = 800 nm. **D)** Number of AAT_Rhg1_ immunogold particles in SCN-penetrated cells relative to the highest number in a similar 2D area of an adjacent normal cell on the same grid (whose quantity is, therefore, 1.0 for each treatment). At least 30 images, from 3 independent experiments, were used to quantify AAT_Rhg1_ immunogold particle abundance for each treatment. Values are mean ± SE. Treatments marked with the same letter were not significantly different (ANOVA, *P* < 0.05).

The abundance of native AAT_Rhg1_ in soybean roots was assessed via standard Western immunoblots using a custom antibody raised against a unique AAT_Rhg1_ peptide sequence (Fig. 1). SCN-infested root regions or analogous regions from mock-inoculated controls were harvested at 4 d postinfection (dpi) from non-transgenic Wm82, Forrest, and Fayette cultivars, which, respectively, carry wild-type (WT) *Rhg1* (*rhg1-c*; single-copy/susceptibility-associated), or *rhg1-a* (3 copies of resistance-associated *Rhg1*), or *rhg1-b* (10 copies of resistance-associated *Rhg1*). Supplemental Fig. S1 confirms antibody recognition of the intended gene product. Figure 1B presents densitometric quantification of the immunoblot band intensities for 4 samples per treatment from 2 independent experiments. In noninfected roots, AAT_Rhg1_ protein abundance was low and similar in WT and low-copy *rhg1-a* samples but consistently greater in high-copy *rhg1-b* samples (Fig. 1, A and B). The detected AAT_Rhg1_ band migrated with an apparent mass of ∼45 kDa, similar to its predicted mass of 46.9 kDa. A significant increase in AAT_Rhg1_ protein abundance was observed in SCN-infected samples of high-copy *rhg1-b* roots (Fig. 1A), about 4.7-fold higher than mock treatment samples of high-copy *rhg1-b* roots (Fig. 1B). Any AAT_Rhg1_ abundance increases were more subtle for susceptible/WT and for low-copy *rhg1-a* roots (Fig. 1, A and B).

Transmission electron microscopy (TEM) immunogold detection experiments were conducted to provide cellular and subcellular resolution regarding AAT_Rhg1_ protein location and relative abundance. Previously, we used similar methods for *Rhg1* locus α-SNAP_Rhg1_ proteins and discovered more than 10-fold greater accumulation of α-SNAP_Rhg1_HC or α-SNAP_Rhg1_LC within syncytium cells (the root cells that comprise the differentiated SCN feeding site), relative to the surrounding cells (Bayless et al. 2016; Bayless et al. 2019). Using the anti-AAT_Rhg1_ antibody to detect native AAT_Rhg1_ in non-transgenic roots during SCN infection, we observed an entirely different pattern (Fig. 1, C and D). Relative to adjacent cells, AAT_Rhg1_ protein abundance was elevated in penetrated root cells along the migration path of SCN. Representative lower-magnification images showing the SCN body, SCN-penetrated root cells, and adjacent normal root cells are shown in Supplemental Fig. S2 and later figures. Using higher magnification, the experiments of Fig. 1 found 4.4-fold to 12.5-fold more immunogold-labeled AAT_Rhg1_ in SCN-penetrated cells relative to a similar area in adjacent normal root cells, across 3 independent experiments. Increases were observed in both SCN-susceptible and 2 types of SCN-resistant cultivars (Fig. 1, C and D). At 3 dpi, anti-AAT_Rhg1_ immunogold particle abundance values relative to adjacent normal cells were lowest for single-copy *Rhg1* (susceptible) roots (∼4.4-fold elevation), moderate for low-copy *rhg1-a* (resistant) roots (∼7.3-fold elevation), and the highest for high-copy *rhg1-b* (resistant) soybean roots (∼11.1-fold elevation). However, at 7 dpi, all genotypes accumulated AAT_Rhg1_ to similar fold-change levels (9.9, 11.0, and 12.5 fold-changes in single-copy, low-copy, and high-copy resistant, respectively). In all cases, the elevated abundance of AAT_Rhg1_ signal relative to nearby non-penetrated root cells within the same microscopy grid was statistically significant (Fig. 1D).

The anti-AAT_Rhg1_ immunogold particles were often associated with vesicles and larger VLB, which were strikingly abundant in the areas of nematode penetration (Fig. 1C; Supplemental Fig. S2; see also additional results below). SCN penetrate root cells and move through the root cortex intracellularly, disrupting the host cells they probe with their stylet and then killing the plant cells they migrate through as they move toward the endodermis. Many of the SCN-adjacent regions with AAT_Rhg1_-bearing vesicles and VLB were observed in deceased recently SCN-penetrated root cortex cells lacking a central vacuole, but subsequent work (presented in subsequent Results sections) showed similar induction of VLB as well as multivesicular bodies (MVB) and paramural bodies in the absence of nematodes in live soybean root and *N. benthamiana* leaf cells expressing AAT_Rhg1_ from strong constitutive promoters. In the experiments summarized in Fig. 1, C and D and Supplemental Fig. S2, the cellular/subcellular morphologies of cells exhibiting elevated AAT_Rhg1_ immunogold signal were similar in the SCN-resistant and SCN-susceptible genotypes.

The AAT_Rhg1_ epitope against which anti-AAT_Rhg1_ was raised is 15 to 20 amino acids from the N-terminus and is predicted by Deep TMHMM to be oriented internally 7 amino acids from the first transmembrane domain, rather than within 1 of the predicted 11 transmembrane domains (https://dtu.biolib.com/DeepTMHMM). AAT_Rhg1_ is predicted to lack a secretion signal peptide (https://services.healthtech.dtu.dk/service.php?SignalP-6.0). Because of the length of primary and secondary antibodies, the immunogold particle can be offset 15 to 30 nm from the location of the bound epitope (Hermann et al. 1996). Hence when AAT_Rhg1_ is membrane-embedded, gold particles labeling AAT_Rhg1_ are expected to variously appear on top of or adjacent to that membrane.

Anti-AAT_Rhg1_ immunogold particles were rarely found in mock treatment samples of all the 3 genotypes tested (Supplemental Fig. S3A). Importantly, in both a susceptible and the 2 types of resistant varieties, anti-AAT_Rhg1_ immunogold particles were also rare in root syncytium cells (which are readily identifiable by the absence of a large vacuole, abundant presence of organelles, and partially degraded cell walls) (Supplemental Fig. S3B).

In control experiments, no specific immunogold labeling could be found on vesicles or other compartments of SCN-penetrated cells when only the secondary antibody was used (Supplemental Fig. S3C). In further control experiments, competitive binding assays were conducted to confirm the antigen specificity of the anti-AAT_Rhg1_ antibody in the context of SCN-penetrated soybean roots imaged by the same EM and immunogold labeling method (Supplemental Fig. S4). In those experiments, the N-terminal 44 amino acid peptide that contains the antigen recognized by our custom AAT_Rhg1_ antibody was purified and preincubated with the AAT_Rhg1_ antibody at a 1-fold or 10-fold molar excess before use on electron microscopy (EM) sections. Multiple adjacent tissue sections from 1 identical region were examined on separate EM grids. The numbers of AAT_Rhg1_ immunolabel gold particles within the same penetrated cells were counted for sections probed with AAT_Rhg1_ antibody pretreated with 1-fold or 10-fold molar excess antigen and compared with the particle numbers for tissue sections probed with AAT_Rhg1_ antibody not pretreated with peptide antigen. Results showed that both 1-fold and 10-fold molar excess of antigen binding significantly reduced the AAT_Rhg1_ immunogold signals within SCN-penetrated cells (Supplemental Fig. S4). This indicates the specificity of the anti-AAT_Rhg1_ immunogold signal for the intended antigen in immunogold-labeled EM soybean root specimens.

**Figure 2.**
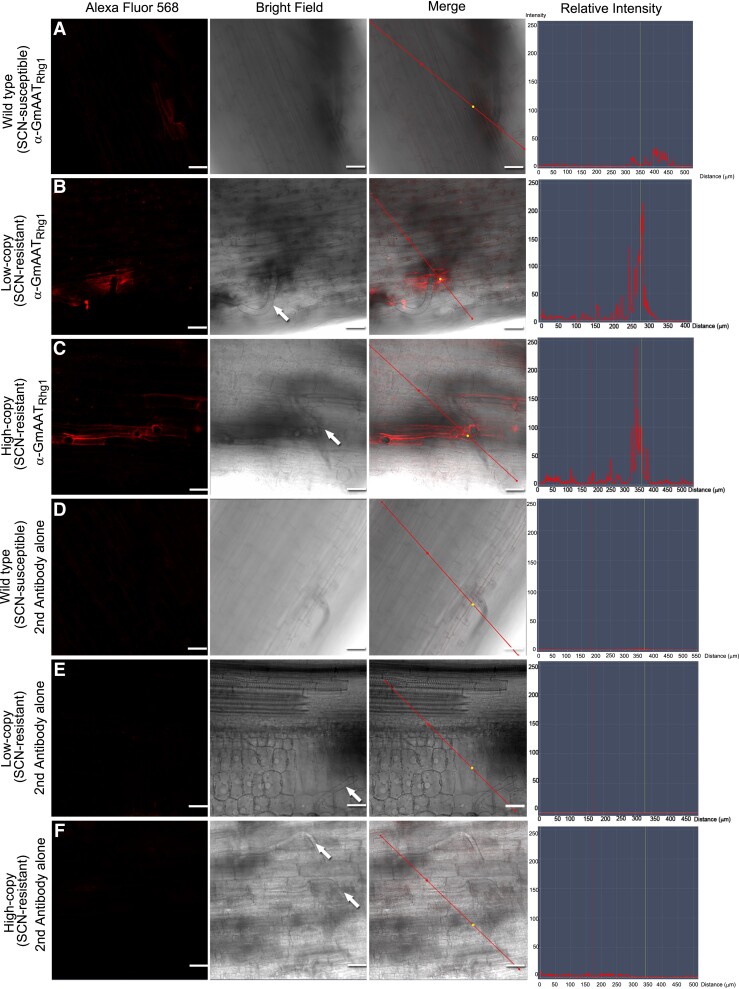
AAT_Rhg1_ abundance increase throughout cells penetrated by SCN. Alexa Fluor 568 column: Representative confocal images of immunofluorescent AAT_Rhg1_ signal 4 d after SCN infestation of roots from WT SCN-susceptible (**A, D)**, *rhg1-a* low-copy SCN resistant (**B, E)**, and *rhg1-b* high-copy SCN resistant (**C, F)** soybean lines. **A–C)** Anti-AAT_Rhg1_ antibody conjugated with secondary antibody, indicating AAT_Rhg1_ localization (fluorescence, left column). **D–F)** Only secondary antibody was used (negative control; imaging sensitivity settings same as **A–C)**. Bright field: DIC images at the same location, collected using T-PMT channel. Nematode bodies are indicated by white arrows. Merge: Overlap of fluorescence and bright-field images. Relative Intensity: Fluorescence signal intensity along the red line shown on merged images. The experiment was repeated on 3 separate dates with similar results. Scale bars = 50 μm.

We further confirmed this discovery of AAT_Rhg1_ abundance elevation in SCN-penetrated root cells using a separate method, confocal microscopy with immunofluorescent detection. This method allows broader visualization of AAT_Rhg1_ distribution within root samples. Roots of non-transgenic SCN-resistant soybean varieties Forrest (*rhg1-a*) and Fayette (*rhg1-b*) were inoculated with 200 J2 SCN per root. Four days after inoculation, SCN-infected root regions were chemically fixed. The in situ location and abundance of native AAT_Rhg1_ protein were monitored by secondary detection of the anti-AAT_Rhg1_ antibody using an Alexa Fluor 568 dye-conjugated anti-rabbit IgG antibody. Under bright-field illumination, the SCN-penetrated cells could be identified readily due to the visible nematode body or the round hole caused by SCN penetration (Fig. 2). Across multiple samples and in 3 independent experiments, confocal fluorescence microscopy detected elevated anti-AAT_Rhg1_ antibody signal only in cells that had been penetrated by a nematode (Fig. 2). Signal was detected throughout the entire cell rather than only at the site of penetration. Labeling of individual vesicles was not evident at the resolution provided in these experiments, although infrequent larger aggregations of fluorescence signal were present. Consistent with the findings of Fig. 1D, AAT_Rhg1_ signal accumulated within SCN-penetrated cells in the roots of SCN-resistant Forrest (*rhg1-a*) and Fayette (*rhg1-b*) plants and also SCN-susceptible Williams 82 (*rhg1-c*) (Fig. 2, A–C). No specific signal was detected near the nematode or in more distal plant cells when secondary fluorescent antibody alone was used (Fig. 2, D–F), serving as a control for nonspecific secondary antibody signal and for sample autofluorescence, both at and away from infection sites.

Taken together, the above results indicate that SCN-penetrated cells undergo a substantial increase in the abundance of the *Rhg1*-encoded amino acid transporter-like protein AAT_Rhg1_. At the early 3 dpi infection stage the level of AAT_Rhg1_ accumulation at SCN-penetrated cells positively correlated with *Rhg1* copy number, with the 10-copy *rhg1-b* soybean variety accumulating the most AAT_Rhg1_. Cells through which SCN migrates are killed so the observed elevation of AAT_Rhg1_ protein abundance is apparently activated in advance of that cell death, possibly as nematodes use their stylet to physically probe the cell that they will next invade and release cell wall-degrading effector proteins (e.g. Siddique et al. 2022).

### AAT_Rhg1_ abundance increase not observed upon wounding with needle

To test whether AAT_Rhg1_ is a wound-inducible protein, roots of Fayette (*rhg1-b*) were penetrated with a 100 µm diameter microneedle. After 3 d, wounded root regions were isolated and chemically fixed. Then, as in the previous section, TEM immunogold labeling experiments and separate confocal microscopy/immunofluorescent detection experiments were performed using the anti-AAT_Rhg1_ antibody. The mechanical damage caused by the microneedle did not elicit signal accumulation in or around the site of needle penetration (Supplemental Fig. S5). Although these experiments do not exclude the possibility that the particular forms or patterns of physical damage caused by the nematode can elicit an elevated abundance of AAT_Rhg1_ signal, the experiments do provide evidence that simple physical penetration of the root cortex is not on its own sufficient to induce elevated abundance of AAT_Rhg1_.

### Abundance of large vesicles is elevated along the migration path of SCN and AAT_Rhg1_ protein accumulates on those vesicles

**Figure 3.**
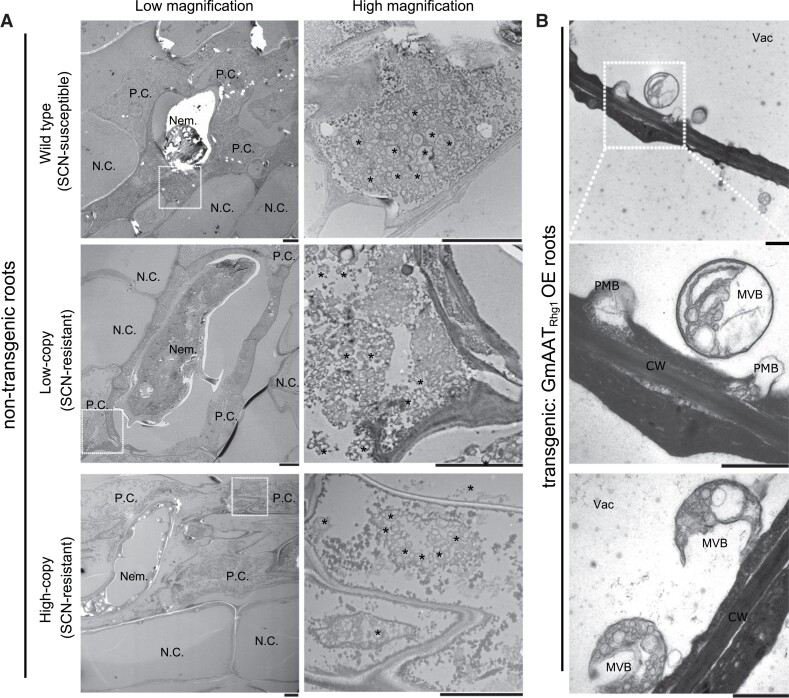
Substantial vesiculation in SCN-penetrated cells in SCN-infested roots of all tested soybean varieties. **A)** Electron micrographs showing root sections of SCN-susceptible (top panels), *rhg1-a* low-copy SCN resistant (middle panels), and *rhg1-b* high-copy SCN resistant (bottom panels) haplotypes. Left column: SCN-penetrated cells (P.C.) surrounding a nematode body (Nem.); 710 × magnification. Right column: further magnification (5600 × images) of the corresponding area indicated by a white dot box on the left. Asterisks mark only a subset of the numerous VLB and MVB observable in SCN-penetrated cells. Additional views of same WT sample are shown in Supplemental Fig. S2. At least 18 fields of view for each genotype were imaged across 3 independent experiments. Nem., nematode; N.C., normal cell; P.C., SCN-penetrated cell. Scale bars = 6 μm. **B)** Representative epoxy-resin TEM images of AAT_Rhg1_ overexpression soybean root cells (elongation region; no SCN infection). An MVB and PMBs in 1 cell (Top); framed area shown at higher magnification (Middle). Two vacuolar MVBs adjacent to tonoplast, containing vesicles of varying sizes (Bottom). CW, cell wall; MVB, multivesicular body; PMB, paramural body; Vac, vacuole. Scale bars = 1 μm.

Independent of immunogold label detection, the above-described TEM images revealed a second observation: a strong increase in the abundance of subcellular vesicles in those cells that had been penetrated by SCN (Fig. 3A; see also Fig. 1C and Supplemental Fig. S2). This increase in vesicle abundance was evident in susceptible roots as well as low-copy *rhg1-a* and high-copy *rhg1-b* soybean roots (Fig. 3A). In addition to numerous vesicles in the ∼50 to 500 nm size range (similar to or larger than common transport or secretory vesicles), some 1 to 2 μm diameter “macrovesicle” VLB were also present. Compared to the adjacent non-penetrated root cortical cells, which retain their large central vacuole, the cells directly surrounding the nematode body showed distinct morphology changes. First, the large central vacuoles were shrunken or otherwise replaced by a nematode body. Second, there were numerous vesicles clustered within the remaining cytoplasm. Third, organelles like mitochondria, ER, or Golgi were rarely observed (Fig. 3A). In all types of soybean roots tested, close inspection of immunogold labeling showed accumulation and co-localization of the AAT_Rhg1_ protein onto those vesicles formed in penetration cells but not in adjacent normal cells (see, for example, Fig. 1C). The results indicate that SCN-penetrated cells undergo a substantial accumulation of VLB and that a substantial proportion of the AAT_Rhg1_ protein accumulates on those vesicles.

Additional TEM experiments were performed using epoxy embedding resin, which generally provides better sample integrity than the LR White resin that is superior for immunodetection. With epoxy-embedded samples, the elevated presence of VLB upon the overexpression of AAT_Rhg1_ was again observed (Fig. 3B and Supplemental Fig. S6). More MVB were also observed, as were paramural bodies (vesicles between the cell membrane and cell wall). MVB structures fused to the cell membrane were observed, apparently releasing the contained vesicles and other content into the apoplast (Fig. 3B and Supplemental Fig. S6). These MVB and other vesicular structures resemble the cellular structures observed in barley responding to powdery mildew infection (An et al. 2006a, 2006b).

### Overexpression of AAT_Rhg1_ in *N. benthamiana* leads to vesiculation with vesicles containing the AAT_Rhg1_ protein

**Figure 4.**
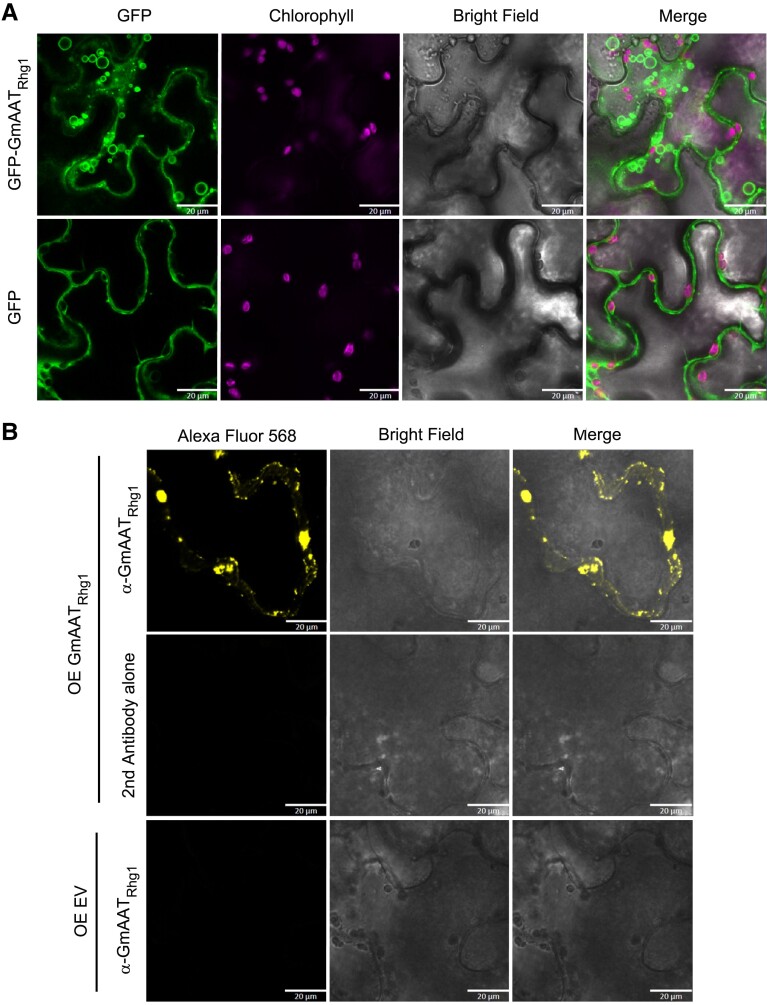
Soybean AAT_Rhg1_ localizes to specialized giant vesicles in *N. benthamiana*. **A)** Top row: GFP-tagged GmAAT_Rhg1_ transiently expressed from a CaMV 35S promoter by agroinfiltration into *N. benthamiana* leaves. Left column: GFP-AAT_Rhg1_ localized on vesicles of sizes ranging from less than 1 μm (arrowhead) to ∼6 μm (arrow). Chlorophyll signal (second column from left) and bright-field image (third column from left) are from the same imaging layer. Merged images (right column) show that the AAT_Rhg1_-containing vesicles were independent of chloroplasts. Lower row: GFP alone expressed similarly as a control. Experiments were replicated on 3 separate dates with similar results. Scale bar = 20 μm. **B)** Immunofluorescent stain confocal imaging showing that untagged GmAAT_Rhg1_ also accumulates in puncta of various sizes. *N. benthamiana* leaves expressing untagged GmAAT_Rhg1_ were immunostained with anti-AAT_Rhg1_ antibody and then probed with a secondary antibody conjugated to Alexa Fluor 568 (Top row). Leaf samples expressing the same GmAAT_Rhg1_ construct immunostained with secondary antibody alone (middle row) or samples expressing empty vector immunostained with both anti-AAT_Rhg1_ antibody and the secondary antibody (bottom row) served as controls. Images were acquired under the same settings across all 3 rows. Untagged soybean AAT_Rhg1_ formed puncta with sizes ranging from less than 1 μm (arrowhead) to ∼6 μm (arrow) (top panels). At least 48 fields of view for each treatment were imaged across 3 independent experiments. Scale bars = 20 μm.

**Figure 5.**
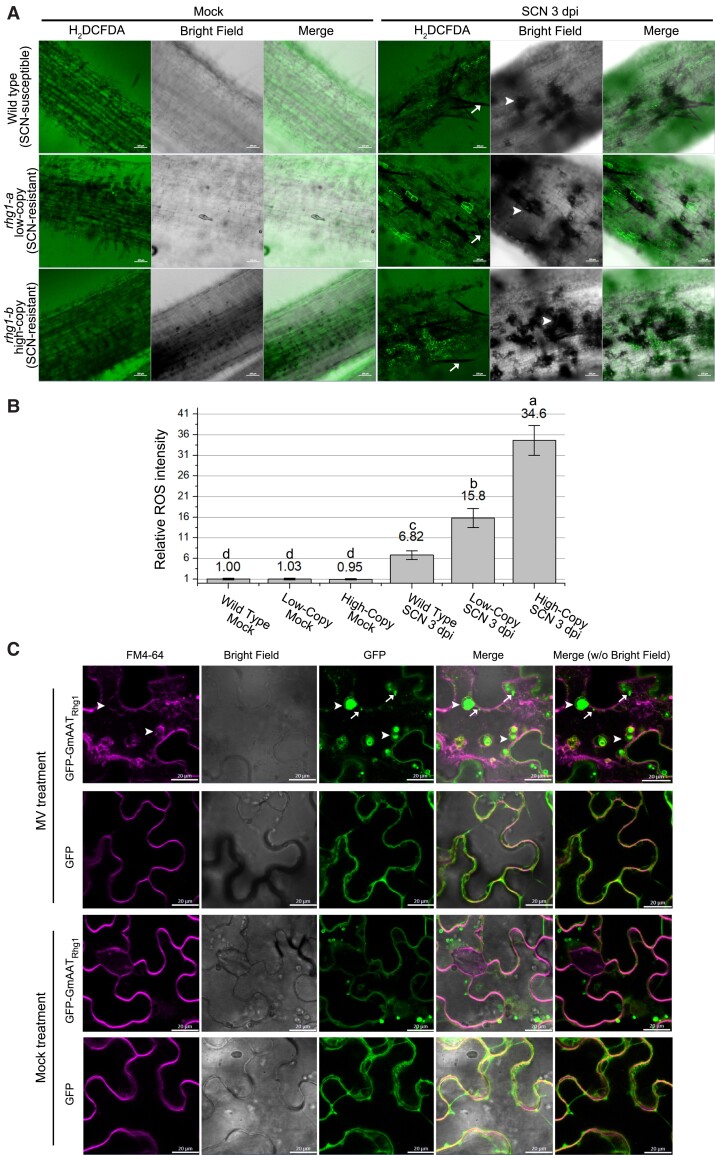
More cells accumulate ROS in the SCN infection zone of resistant roots and ROS stress leads to endocytosis-associated fusion of AAT_Rhg1_-containing vesicles. **A)** Representative 3 dpi confocal images of mock-treated (left) and SCN-infected (right) soybean roots carrying WT (top row), *rhg1-a* (middle row), and *rhg1-b* (bottom row) haplotypes, after incubation in H_2_DCFDA. Bright fluorescent pixels in H_2_DCFDA channel report endogenous ROS production; bright-field images are shown in grayscale. Arrows with stem point to representative SCN body; arrowheads point to representative lesions caused by SCN infection. Scale bars = 100 μm. **B)** Quantification of ROS-producing cells in soybean roots with or without SCN infection as in **(A)**. Relative extent of ROS production was calculated by the total H_2_DCFDA fluorescent area divided by the total root cell area with fluorescent background and then normalized to WT mock control treatment. For each treatment, at least 16 images of 8 independent roots from 2 independent experiments were used for calculation. Error bars indicate the standard error of the mean. Treatments with the same letter are not significantly different (ANOVA, *P* < 0.05). **C)** Representative confocal images showing, upon cellular ROS stress, AAT_Rhg1_-containing vesicles fused into larger vesicles through an endocytosis pathway in *N. benthamiana* leaf cells expressing GFP-AAT_Rhg1_ (top row) but not in cells expressing GFP control (second row). MV treatment: methyl viologen (inducer of superoxide and other ROS). Transiently transformed *N. benthamiana* leaves were treated with 20 μm MV or mock treatment for 8 h, then stained with the endocytic tracer FM4-64 30 min prior to confocal imaging. Arrows with stem point to representative puncta; larger arrowheads point to representative larger giant vesicle-like agglomerations of GFP-AAT_Rhg1_. Areas of overlap between the magenta and green signal are lighter (more white) in the merged image (right column). At least 27 fields of view were imaged for each treatment across 3 independent experiments. Scale bars = 20 μm.

Expression of AAT_Rhg1_ protein increases along the SCN infection path. As one means of investigating impacts of AAT_Rhg1_ protein accumulation in planta, we expressed soybean AAT_Rhg1_ in *N. benthamiana*. N-terminally GFP-tagged AAT_Rhg1_ or a GFP-only control, driven by a double CaMV 35S promoter, were transiently expressed in *N. benthamiana* leaves by agroinfiltration. Seventy-two hours after agroinfiltration, the localization of GFP-AAT_Rhg1_ was analyzed by confocal microscopy. The fluorescence signal for both GFP-AAT_Rhg1_ and GFP was readily detectable over background. Interestingly, in addition to its distribution throughout the plasma membrane, GFP-AAT_Rhg1_ was present in the form of multiple primarily cytoplasmic puncta (small spots) as well as large hollow (peripherally fluorescent) or solidly green fluorescent vesicle-like structures (Fig. 4). These large hollow vesicles were of various sizes from ∼0.58 to 5.5 μm diameter, and clearly did not overlap with chloroplasts (Fig. 4A). The structures are analogous in size and shape to the VLB and MVB we observed in soybean roots using EM. The elevated abundance of large VLB and MVB-like structures was also analogous to the elevated abundance observed in soybean cells in the SCN penetration zone. As expected, the GFP-only control localized diffusely throughout the cytoplasm and, unlike GFP-AAT_Rhg1_, did not cluster in puncta or small spheres. In these and other *N. benthamiana* experiments with AAT_Rhg1_ (see below), bright-field images of the GFP-only control samples showed typical healthy leaf cell cytoplasmic configuration while the cytoplasm of GFP-AAT_Rhg1_ samples frequently contained a more granular appearance (Fig. 4A and Fig. 5C).

We conducted additional immunolocalization analyses using untagged AAT_Rhg1_. *N. benthamiana* leaves transiently expressing soybean AAT_Rhg1_ or empty vector were chemically fixed at 72 hpi. Samples were incubated with the anti-AAT_Rhg1_ antibody followed by secondary incubation with Alexa Fluor 568 dye for immunofluorescence confocal microscopy. Other samples expressing AAT_Rhg1_ were incubated with secondary Alexa Fluor 568 dye alone as a control. AAT_Rhg1_ immunofluorescent signal was detected in cells expressing AAT_Rhg1_ and not in empty vector controls (Fig. 4B). The signal was specific to the primary anti-AAT_Rhg1_ antibody as the secondary antibody alone control did not show any signal (Fig. 4B). Interestingly, the specific immunofluorescent signal again accumulated in puncta spots and in vesicle-like structures with an apparent size ranging from 0.94 to 6.6 μm. The free/diffuse cytosolic signal in AAT_Rhg1_ samples was less prominent, as was also observed for GFP-AAT_Rhg1_ but not for the GFP controls of Fig. 4A.

To associate the observed GFP-AAT_Rhg1_-containing VLB with defined cellular structures, we used confocal laser scanning microscopy to test for co-localization of GFP-AAT_Rhg1_ and 5 organelle markers in *N. benthamiana* leaves (Nelson et al. 2007) (Supplemental Fig. S7). GFP-AAT_Rhg1_ was co-expressed with RFP-tagged markers for Golgi, ER, plasma membrane, plastids, or peroxisomes. Upon coexpression with each of the organelle markers, GFP-AAT_Rhg1_ displayed the same VLB localization that GFP-AAT_Rhg1_ alone showed in Fig. 4. Partial co-localization was observed for GFP-AAT_Rhg1_ and the ER marker, and for GFP-AAT_Rhg1_ and the plasma membrane marker (Supplemental Fig. S7). GFP-AAT_Rhg1_ did not show co-localization with the Golgi, plastid, or peroxisome markers (see also Supplemental Fig. S8).

Together, the immunofluorescence and green fluorescent-protein tagging showed that, as in soybean root cells along the SCN migration path, AAT_Rhg1_ overexpressed in *N. benthamiana* leaves accumulates on profuse vesicles and VLB.

### H_2_O_2_ accumulates in some cells around the SCN at the early infection stage

ROS signaling can be a mediator of plant defenses against pathogens and ROS production in roots during cyst nematode infections has been studied using multiple approaches (see Introduction). We examined the spatial pattern of ROS accumulation as SCN migrates through roots. ROS production was monitored in susceptible and in *rhg1-a* and *rhg1-b* SCN-resistant soybean varieties, by tracking bright green fluorescence from the hydrogen peroxide probe 2′,7′-dichlorodihydrofluorescein diacetate (H_2_DCFDA). Roots were examined at 3 d postinfection, a time at which nematode migration is still occurring but some nematodes have initiated feeding and other infections have terminated. We found that H_2_O_2_ was induced by SCN in root cells of all the 3 types tested, but not in the mock treatments (Fig. 5). Interestingly, only a portion of root cells around the SCN (indicated by arrows in Fig. 5A) had strong H_2_DCFDA fluorescent signals. Scattered lesions (dead root cells) in the area of nematode invasion are commonly observed a few days after initial root exposure to SCN, but neither the lesions (arrowhead in Fig. 5A) nor the SCN (arrow with stem) showed elevated ROS signals. Reproducibly, there were more cells with positive H_2_DCFDA fluorescent signals in the *rhg1-b* high-copy variety than the *rhg1-a* low-copy variety or the WT. Compared with the WT mock treatment, the percent root area with ROS accumulation was the lowest for WT single-copy *Rhg1* (susceptible) roots (∼6.8-fold elevation), moderate for low-copy *rhg1-a* (resistant) roots (∼15.8-fold elevation), and the highest for high-copy *rhg1-b* (resistant) soybean roots (∼34.6-fold elevation) (Fig. 5B). There were no significant differences between all the mock treatments of the 3 varieties (Fig. 5B).

### ROS accumulation enhances endocytosis-associated accumulation of AAT_Rhg1_-containing vesicles

Because soybean roots present a challenging experimental system for transient gene expression and for confocal imaging, *N. benthamiana* leaves were used to initiate the investigation of the potential interaction of AAT_Rhg1_ with ROS. *N. benthamiana* leaves transiently expressing GFP-AAT_Rhg1_ or GFP alone as a control were infiltrated with 20 μm methyl viologen (MV; paraquat). MV is an inhibitor of photosynthetic electron transport chains that induces the elevation of ROS in plant cells (Han et al. 2015). Eight hours after MV treatment, the leaf apoplast was infiltrated with FM4-64 and then imaged 30 min later. Confocal live imaging of FM4-64 dye uptake is a standard technique to monitor vesicle dynamics in endocytic pathways (Bolte et al. 2004). As noted above, expression of GFP-AAT_Rhg1_ (in the absence of MV) led to the accumulation of green fluorescent puncta as well as green fluorescent vesicle-like structures (Fig. 5C and Supplemental Fig. S9, GFP column of images). Across independent samples and experiments, expression of GFP-AAT_Rhg1_ in the presence of MV consistently led to the accumulation of more and larger green fluorescent vesicle-like structures with diameters of approximately 6 to 10 μm (arrowheads, Fig. 5C). Within some large vesicles (MVB) multiple small round vesicles with GFP-AAT_Rhg1_ fluorescent signals were present, suggesting that the larger vesicles had formed through uptake of multiple small vesicles.

The FM4-64 channel (red fluorescence) in these Fig. 5C experiments also consistently showed abundant accumulation of spherical vesicle/VLB/MVB-like structures in the GFP-AAT_Rhg1_ + MV samples. FM4-64 labels membranes independent of the presence or absence of GFP-AAT_Rhg1_. At the imaged time point 30 to 40 min. after dye application, FM4-64-stained membranes would primarily but not exclusively have recent endocytic origins.

Merging of FM4-64 and GFP images showed that FM4-64-stained membranes colocalized with the fused large vesicles (indicated by GFP fluorescence, arrowheads, Fig. 5C and Supplemental Fig. S9) but not with smaller GFP-AAT_Rhg1_-containing vesicles (arrows, Fig. 5C). This suggests that endocytic vesicles fuse with and possibly promote the agglomeration of GFP-AAT_Rhg1_ into larger clusters. The small GFP-AAT_Rhg1_ vesicles may have formed prior to the addition of FM4-64 or might not be derived from endocytic events.

To reiterate, we found in *N. benthamiana* that upon MV-induced oxidative stress, vesicle-associated AAT_Rhg1_ was more often associated with larger VLB and MVB. These larger vesicles could be stained by a 30-min FM4-64 treatment, indicating that this fusion is associated with an endocytic internalization process. This endocytic vesiculation response to ROS stimuli, which was observed in the presence of elevated AAT_Rhg1_ expression (Fig. 5), was reminiscent of our electron micrograph discoveries that SCN-penetrated cells in soybean roots form larger AAT_Rhg1_-containing VLB and MVB that can contain multiple small AAT_Rhg1_-containing vesicles (Figs. 1C and 3).

### AAT_Rhg1_ interacts on vesicles with GmRBOHG, an SCN-responsive NADPH oxidase homolog

**Figure 6.**
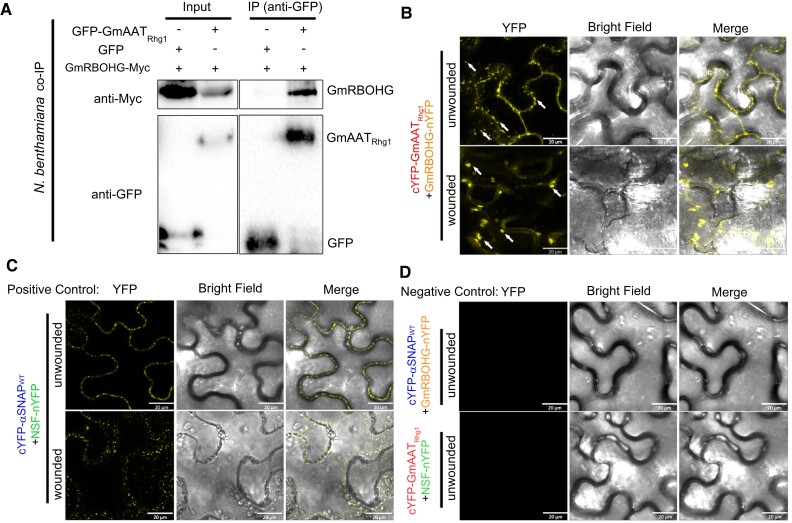
AAT_Rhg1_ and GmRBOHG interact in plants and wound treatment changes their interaction sites from small to large vesicles. **A)** Immunoblots showing Co-IP of AAT_Rhg1_ with soybean RBOHG. Myc tagged GmRBOHG was co-expressed with GFP-AAT_Rhg1_ in *N. benthamiana* leaves by agroinfiltration. GmRBOHG co-expressed with GFP alone served as a negative control. IP (anti-GFP): leaf lysates harvested at 72-h post-infiltration were immunoprecipitated with anti-GFP beads and immunoprecipitates were assessed by immunoblotting using anti-Myc (right column, top panel) and anti-GFP antibodies (right column, bottom panel). Input: the presence of GmRBOHG-Myc and GFP-AAT_Rhg1_ or GFP in total protein for each treatment was confirmed (left blots). **B)** Representative images from BiFC assays showing the localization of AAT_Rhg1_ interaction with GmRBOHG in plants, with or without wounding treatment. GmRBOHG-nYFP was co-expressed transiently with cYFP-AAT_Rhg1_ in *N. benthamiana* leaves under unwounded conditions or wounded conditions (top 2 panels). As a positive control, GmNSF-nYFP and cYFP-α-SNAP_Rhg1_WT were co-expressed similarly (middle 2 panels). As a negative control, the same constructs were co-expressed with different pairings (bottom 2 panels). YFP fluorescence indicated by yellow color (left column) was detected from epidermal cells. In cells coexpressing cYFP-AAT_Rhg1_ and GmRBOHG-nYFP, complemented fluorescence signal was detected in small vesicles (indicated by white arrows in the first panel) in the unwounded condition, or in large vesicles (white arrows in the second panel) in wounded cells. The experiments were repeated on 3 separate dates with similar results. Scale bars = 20 μm.

We hypothesized that AAT_Rhg1_ physically interacts with 1 or more previously discovered SCN pathogenesis or ROS-associated proteins, and used *N. benthamiana* agroinfiltration and co-immunoprecipitation (co-IP) to conduct planta tests for interactions. GmRBOHG, the *Rhg1*-encoded α-SNAP_Rhg1_, and WI12_Rhg1_ proteins and 8 SCN effectors reported to be expressed during SCN infection (Pogorelko et al. 2020) were tested for interaction with AAT_Rhg1_. Only GmRBOHG gave a positive result in the preliminary prescreening, and it was then confirmed upon further testing to be an AAT_Rhg1_ interactor (Fig. 6). *Glyma.06G162300*, which encodes GmRBOHG (Ranjan et al. 2018), is a soybean ortholog of the gene encoding defense-associated Arabidopsis RBOHD (Miller et al. 2009; Li et al. 2014; Lee et al. 2020). In previous genome-wide expression profiling, *Glyma.06G162300* transcript abundance was significantly upregulated after SCN infection in both susceptible and resistant lines, with greater abundance in resistant lines at 3 dpi (Wan et al. 2015; Liu et al. 2019). We co-expressed epitope-tagged GmRBOHG-Myc with GFP-tagged AAT_Rhg1_ (or GFP-only control) in *N. benthamiana* leaves. GmRBOHG-Myc co-immunoprecipitated with GFP-AAT_Rhg1_ but not with GFP alone (Fig. 6A).

To validate these results and to investigate the cellular location of GmRBOHG-AAT_Rhg1_ interaction, bimolecular fluorescence complementation (BiFC) experiments were carried out. Stringent positive and negative controls are necessary for BiFC experiments (Kudla and Bock 2016); we used the known protein interaction partners NSF and α-SNAP_Rhg1_WT of soybean (Bayless et al. 2016) for this purpose. NSF and α-SNAP interact in vitro and in vivo, where they participate in the disassembly of SNARE protein bundles that are associated with vesicle trafficking, including at the plasma membrane (Zhao et al. 2015). Here, AAT_Rhg1_ with an N-terminal cYFP tag (cYFP-AAT_Rhg1_) was transiently co-expressed in *N. benthamiana* leaves with either GmRBOHG-nYFP or the negative control NSF-nYFP. cYFP-α-SNAP_Rhg1_WT was co-expressed with GmRBOHG-nYFP to serve as another negative control. cYFP-α-SNAP_Rhg1_WT and NSF-nYFP were co-transformed within the same leaf as the above samples to serve as a positive expression control for the negative controls (Kudla and Bock 2016). We observed positive interaction signals, indicated by yellow fluorescence, only upon coexpression of cYFP-AAT_Rhg1_ and GmRBOHG-nYFP, and for the positive control cYFP-α-SNAP_Rhg1_WT + NSF-nYFP (Fig. 6B). Interestingly, the signals for interaction between cYFP-AAT_Rhg1_ and GmRBOHG-nYFP were localized on small vesicle-like puncta within the cytoplasm, which was consistent previous AAT_Rhg1_ localization results (Figs. 1C and 4). The known vesicle trafficking contributors α-SNAP and NSF also interacted in vesicle-like puncta in our BiFC assay (Fig. 6B). The other control combinations did not give yellow fluorescence under the same confocal detection settings (Fig. 6B).

A substantial portion of the observed AAT_Rhg1_ localized onto larger fused vesicles under MV-induced ROS stress when overexpressed in *N. benthamiana* leaves (Fig. 5C; see also Figs. 1C and 4A for soybean). To test whether the co-localization pattern of AAT_Rhg1_/GmRBOHG changed under similar stress, a hemostat wounding method was used after 60 h co-expression of cYFP-AAT_Rhg1_/GmRbohG-nYFP or cYFP-α-SNAP/NSF-nYFP control. After wounding, the leaves were left for 30 min in the air before confocal microscopy. After this treatment, the reconstituted YFP signal indicating the interaction of cYFP-AAT_Rhg1_ and GmRbohG-nYFP was shifted toward larger vesicles (Fig. 6B). The α-SNAP/NSF interaction signal remained on similarly sized vesicles with or without wounding treatment (Fig. 6B). As a point of clarification, the microneedle experiments of Supplemental Fig. S5 tested if punctures would mimic nematode penetration and cause AAT_Rhg1_ protein abundance increases, in soybean roots with untagged AAT_Rhg1_ expressed from its native locus. The present experiments (Fig. 6B) used constitutively expressed AAT_Rhg1_ in *N. benthamiana* leaves and tested if its interaction with GmRBOHG changes in the presence of the elevated ROS and other stresses caused by wounding. The experiment indicated that the ROS-generating GmRBOHG protein, previously shown to be transcriptionally upregulated during SCN infection, interacts with AAT_Rhg1_ in small vesicles in normal conditions and in larger vesicles after wounding.

### Simultaneous elevation of AAT_Rhg1_ and GmRBOHG abundance causes ROS production

**Figure 7.**
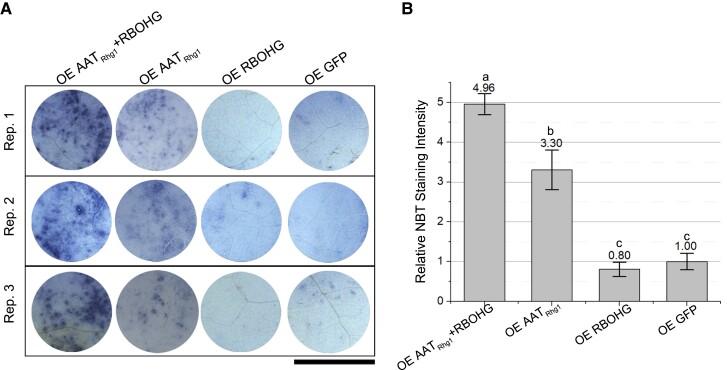
Simultaneous overexpression of GmRBOHG and AAT_Rhg1_ causes increased superoxide production in *N. benthamiana* leaves. **A)** Representative images of *N. benthamiana* leaf regions stained with NBT 72 h. after agroinfiltration to overexpress (OE) (from left to right) GmRBOHG and AAT_Rhg1_ protein, AAT_Rhg1_ alone, GmRBOHG alone, or GFP control protein alone. Images from 3 biological replicates from separate dates are shown. Within each row, the leaf sectors shown are all from the same leaf and were photographed at the same time as part of a single image. Scale bar = 1 cm and applies to all images. **B)** Quantification of NBT staining intensity of transformed leaf regions described in **(A)**. Total area with NBT staining was measured using ImageJ and divided by total infiltration area, and normalized to the results for GFP alone control within the same replicate. *n* = 12 plants, mean ± SE are shown, and treatments with the same letter are not significantly different (ANOVA, *P* < 0.05).

Having discovered that AAT_Rhg1_ and GmRBOHG physically interact in planta, experiments were then carried out to determine if AAT_Rhg1_ alters ROS generation in concert with GmRBOHG (Fig. 7). GmRBOHG and AAT_Rhg1_ without epitope tags were co-expressed in *N. benthamiana* leaves under control of CaMV 35S promoters. GmRBOHG alone, AAT_Rhg1_ alone, or GFP alone were expressed in the same leaf within the same biological replicate to serve as controls; 72 h after agroinoculation, leaves were detached, stained with nitroblue tetrazolium (NBT) for one-half hour, and then destained. NBT is a standard histochemical stain that detects superoxide (Beauchamp and Fridovich 1971). Numerous NBT-positive spots were detected when coexpressing GmRBOHG and AAT_Rhg1_ or expressing AAT_Rhg1_ alone, while within the same leaves few or no NBT-positive spots could be seen in the cells transiently expressing GmRBOHG alone, or GFP (Fig. 7A). Quantification of staining areas confirmed a significant elevation of ROS production when expressing AAT_Rhg1_ alone. There was even more ROS production when GmRBOHG and AAT_Rhg1_ were co-expressed (Fig. 7B). GmRBOHG and AAT_Rhg1_ synergize to promote more ROS production than either protein causes when overexpressed on its own.

## Discussion

The *Rhg1* locus has for multiple decades been the primary means of control of the most economically damaging pathogen of soybean, SCN, but little is known about how AAT_Rhg1_ contributes to resistance (Mitchum 2016; Yan and Baidoo 2018). One of the main findings of the present study is that AAT_Rhg1_ protein levels increase approximately 10-fold along the path of SCN root invasion. The hypothesis that AAT_Rhg1_ protein abundance differences are a determinant of resistance had previously been proposed (Cook et al. 2012, 2014), because unlike the α-SNAP_Rhg1_ proteins, there are no amino acid polymorphisms in AAT_Rhg1_ between resistant and susceptible varieties. Instead, *Glyma.18G022400* mRNA abundance in noninfected tissues had been shown to scale with *Rhg1* locus copy number, showing significant elevation in multicopy *Rhg1* SCN-resistant genotypes (Cook et al. 2014; Wan et al. 2015). Moreover, whole-genome expression profiling reported that *Rhg1-GmAAT* mRNA abundance is elevated in SCN-infected root tissues (Kandoth et al. 2011; Matsye et al. 2011). By 7 dpi we observed similar fold-change increases in AAT_Rhg1_ protein abundance, relative to neighboring cells, in both SCN-susceptible and SCN-resistant genotypes. However, at 3 d after SCN infection the fold-change increase of AAT_Rhg1_ protein in cells along the SCN migration path, relative to nearby noninfested cells, scaled with *Rhg1* locus copy number. This was on top of the baseline (non-inoculated root) elevation of AAT_Rhg1_ protein abundance in roots carrying the higher copy numbers of the *Rhg1* locus. *Rhg1* copy number has also been shown to positively correlate with SCN resistance efficacy, especially when isolated from contributions from other loci such as *Rhg4* (Cook et al. 2014; Yu et al. 2016; Patil et al. 2019). The differences in AAT_Rhg1_ protein abundance and extent of elevation at infection sites are a likely mechanism contributing to the documented differences in resistance efficacy between *Rhg1* haplotypes.

Equally or more striking, the present study discovered that AAT_Rhg1_ protein specifically accumulates along the SCN root migration path, relative to its abundance in the root cells a few cells away from the migration path or in any other observed root cells. Most SCN-penetrated root cells would be dead or dying at the time of fixation for microscopy, but they apparently had been stimulated to express elevated levels of AAT_Rhg1_ protein prior to that event. One reason that this finding is of interest is because α-SNAP_Rhg1_, the protein product of the adjacent gene within the same *Rhg1* locus, was previously shown to accumulate more than 10-fold specifically within the syncytium cells that, in SCN-susceptible genotypes, serve for a few weeks as the biotrophic interface for cyst nematode feeding (Bayless et al. 2016; Bayless et al. 2019). We observed little or no AAT_Rhg1_ immunogold signal within syncytia for all 3 of the soybean varieties tested. This indicates that the resistance-contributing genes within the *Rhg1* locus not only encode distinctly different proteins, but those proteins also appear likely to act in SCN defense at spatially and temporally separate locations in the infection court. The multi-decade durability of *Rhg1*-encoded SCN resistance may have been due in part to the lower evolutionary potential of SCN relative to some microbial plant pathogens (McDonald and Linde 2002). However, the present study provides experimental findings supporting the hypothesis that the durability of *Rhg1* is enhanced because it is a naturally occurring “resistance stack” encoding more than 1 mode of action.

The mechanism through which AAT_Rhg1_ activates defenses has remained unknown. As noted in the Introduction, collaborators (A. Reinders, J.M. Ward, B.E. Broeckling, and D.R. Bush, unpublished data) tested AAT_Rhg1_ for amino acid transport activity in 2 heterologous model systems (*Xenopus* oocytes and yeast) previously used by those groups to successfully describe plant amino acid transporters. No activity was detected for 29 amino acids and other compounds, possibly because AAT_Rhg1_ was not functionally expressed. Alternatively, AAT_Rhg1_ may transport a different substrate than the compounds tested. None of the *Rhg1* genes encodes an NB-LRR, RLK, or other protein type that commonly serves the role of pathogen detection and defense activation in plants (Dangl and Jones 2001). Plants sense infection in 1 cell and then can activate defenses in nearby noninfected cells and/or systemic cells using a variety of mediators, including glutamate, ROS, Ca^2+^, salicylic acid, and *N*-hydroxypipecolic acid, to name just a few examples (Bernsdorff et al. 2016; Hartmann et al. 2018; Toyota et al. 2018; Wang et al. 2019). Local stimuli such as insect herbivory can activate glutamate as a longer-distance wound signal to rapidly initiate defense responses in undamaged parts (Toyota et al. 2018). ROS and electrical signaling, mediated by RBOH proteins and glutamate receptor-like proteins, control distal activation of JA signaling during tomato responses to root-knot nematodes (Wang et al. 2019). Those examples may be germane because AAT_Rhg1_, while not shown to be a glutamate transporter, was recently reported to increase tolerance to toxic levels of exogenously supplied glutamate, and it impacted glutamate abundance and transport (Guo et al. 2019).

Our testing revealed the physical association of the AAT_Rhg1_ and GmRBOHG proteins in planta. RBOHG was chosen from the 17 soybean RBOH genes because it is an ortholog of Arabidopsis RBOHD and was the only homolog significantly upregulated in an SCN-resistant variety 3 d after SCN infection (Wan et al. 2015; Ranjan et al. 2018; Liu et al. 2019). We further observed in *N. benthamiana* that overexpression of GmRBOHG did not elevate ROS, overexpression of AAT_Rhg1_ was sufficient to raise ROS levels above background, but co-expression of both proteins caused significantly more elevation of ROS. In soybean, we found that AAT_Rhg1_ abundance increases along the path of nematode invasion and found greater 3 dpi increases in ROS in resistant haplotypes. GmRBOHG mRNA abundance has been shown to increase in SCN-infected tissue (Wan et al. 2015; Liu et al. 2019). The present work hence establishes as a future priority a dissection of cause-effect relationships between AAT_Rhg1_/GmRBOHG interaction, ROS elevation, and glutamate elevation in the activation of defense responses during cyst nematode infections.

Another intriguing feature of the present study was the extensive vesicle and VLB (macrovesicle) production observed along the SCN migration path in roots, and the association of AAT_Rhg1_ with extensive vesicles and VLB both in *N. benthamiana* and along the SCN infection path in soybean. In split-YFP experiments, GmRBOHG interaction with AAT_Rhg1_ was primarily observed on these vesicles.

When considering the above and other findings about these AAT_Rhg1_-induced/AAT_Rhg1_-carrying vesicles, the vesicles do have things in common with the MVB and paramural bodies observed, for example, in barley responding to powdery mildew infection (An et al. 2006a, 2006b; Li et al. 2018), and with the extracellular vesicles (EVs) recently found to play prominent roles during plant-microbe interactions (Rutter and Innes 2017). First, the vesicular structures we observed were not common in normal cells—their presence was elicited by pathogen infection. Second, they are physically close to the penetration structure, for example, the nematode body (this study) or the haustoria structure (plant-fungal interaction). EVs that accumulate during microbial pathogen invasion have been shown to be defense cargo shuttle vectors, carrying various defense-related proteins, siRNAs, and lipid signals that play roles in plant defense responses (Rybak and Robatzek 2019). In Arabidopsis, defense-related sRNAs could be shuttled into the necrotrophic fungus *Botrytis cinerea* via EVs (Cai et al. 2018). The AAT_Rhg1_-associated vesicles that we observed may have similar roles during SCN infection. However, we have only preliminary evidence (Supplemental Fig. S6) that the vesicles and VLB might be exported across the cell plasma membrane. For most of the present study, they were observed within penetrated soybean root cells, or within *N. benthamiana* cells overexpressing AAT_Rhg1_, or in soybean root apoplastic fluid surrounding the nematode migration path which includes intracellular remnants from recently deceased penetrated root cells. However, in *N. benthamiana* FM4-64 experiments that monitored uptake of externally labeled plasma membrane, at least some of the AAT_Rhg1_-bearing vesicles were associated with endocytic rather than exocytic processes. In *N. benthamiana* overexpressing AAT_Rhg1_, co-localization of AAT_Rhg1_ with a plasma membrane marker was observed on internally localized macrovesicles as well as the plasma membrane. Hence at least some of the observed AAT_Rhg1_-containing vesicles may be endocytic vesicles, reminiscent of those that become more abundant as a transcytosis precursor to papilla formation or when RLKs such as FLS2 have been activated for signaling (Geldner and Robatzek 2008; Beck et al. 2012; Nielsen and Thordal-Christensen 2013; Li et al. 2018).

It bears mention that our AAT_Rhg1_ protein localization findings, with native protein expressed from the native gene in soybean roots or with native or GFP-tagged protein expressed in *N. benthamiana* leaves, were internally consistent but differed from the observation of Guo et al. (2019). In limited work, they expressed a GFP-tagged AAT_Rhg1_ in tobacco and observed plasma membrane localization but also some GFP signal in the nucleus (which resembled the nuclear localization of free GFP in their figure). The resolution of that figure was too low to detect vesiculation, but tobacco may also be less sensitive to the expression of soybean AAT_Rhg1_.

Discovery of physical and functional interactions between AAT_Rhg1_ and RBOHG is perhaps less surprising given the importance of ROS signaling in plant responses to nematode infection. Primarily defensive roles have been established in responses to root-knot nematodes (e.g. Melillo et al. 2006; Zhou et al. 2018; Wang et al. 2019; Chen et al. 2020). The impacts of ROS accumulation during plant responses to cyst nematodes are subtle. ROS production contributes to nematode virulence but also apparently contributes to plant resistance, indicating the probable importance of the level, timing, and location of ROS production (Waetzig et al. 1999; Kandoth et al. 2011; Siddique et al. 2014; Chen et al. 2020; Lakhssassi et al. 2020b; Chopra et al. 2021). Production of ROS scavenging enzymes is also induced, reinforcing the concept that complex homeostatic mechanisms are at play (Kandoth et al. 2011; Chen et al. 2020; Lakhssassi et al. 2020b). Potential engagement of the serine hydroxymethyltransferase *Rhg4* gene product GmSHMT08 in ROS homeostasis (as well as in interaction with the *rhg1-a* α-SNAP_Rhg1_LC protein) has also been noted (Lakhssassi et al. 2020a, 2020b).

Detailed studies of the mechanisms that lead to ROS generation in soybean-SCN interactions are not available. We speculate, as one possibility, that the SCN-induced VLB may provide a longer-lived membrane site for the ROS generation machinery. Within cells damaged by nematode penetration, these types of vesicles could serve as a briefly enduring cellular compartment where those defense responses can continue to function. A similar concept has been presented by Klink and colleagues, who proposed (Pant et al. 2015) that a transiently protected living plant cell could secrete materials in the vicinity of the nematode to disarm it, prior to that plant cell succumbing to its targeted demise. We observed that GmRBOHG physically interacts with AAT_Rhg1_ within puncta that resemble these VLB. Upon SCN infection, the accumulation of AAT_Rhg1_ could recruit upregulated GmRBOHG onto those VLB through their interaction. Alternatively, AAT_Rhg1_ may activate RBOHG and other respiratory burst oxidase homologs at the cell membrane, and then end up on VLB simply as a recycling mechanism. The yellow puncta observed in Fig. 6B and Supplemental Fig. S8 are not colocalized with peroxisomes. We further note that the SCN penetration through the root and secretion of plant cell wall-degrading enzymes is likely to release damage-associated molecular patterns (DAMPs) that activate PTI, including ROS production (Siddique et al. 2022). AAT_Rhg1_ may play a supplementing/amplifying role in strengthening DAMP-induced PTI. Furthermore, in the present study, simple root puncture with a microneedle did not elevate AAT_Rhg1_ protein abundance. Other signals, yet to be discovered, mediate the elevation of AAT_Rhg1_ protein abundance. Regardless, the elevated coexpression of AAT_Rhg1_ and GmRBHOG did enhance ROS production, which may be a key early defense against SCN infection that directly weakens the nematode and/or signals to neighboring cells to potentiate defenses.

It remains possible that defense-related functions other than ROS generation are also mediated by the observed AAT_Rhg1_-containing vesicles. For example, the Peking-type SCN resistance requires *rhg1-a* and *Rhg4*. *Rhg4* encodes a serine hydroxymethyltransferase that interconverts serine and glycine, essential for cellular 1-carbon metabolism. The amino acids transported by AAT_Rhg1_ are not known and may include potential substrates for the Rhg4 hydroxymethyltransferase. These remain as topics for future study.

Taken together, the present study reports distinct tissue and subcellular sites of the elevated abundance of the putative amino acid transporter AAT_Rhg1_, along the path of SCN infection. AAT_Rhg1_ expression is associated with the accumulation of vesicles and VLB, and with activities that elevate ROS production, revealing mechanisms of the successful *Rhg1*-mediated SCN resistance that might be applied to other plant-nematode interactions.

## Materials and methods

The methods utilized are described in detail in the Supplemental Methods S1. The materials and data are available upon request.

### Nematode inoculum

Surface-disinfested J2 SCN of HG type 0 were utilized as inoculum.

### Plasmid constructs

Transient overexpression of soybean (*Glycine max*) AAT_Rhg1_ and GmRBOHG utilized the respective ORF with a double CaMV 35S promoter with TMV omega enhancer and NOS terminator. BiFC vectors and co-IP vectors utilized single CaMV 35S promoters.

### Transgenic soybean root and *N. benthamiana* experiments

Transgenic Williams 82 soybean roots were generated using *Agrobacterium rhizogenes* ArQua1 strains as described (Melito et al. 2010). Proteins were transiently expressed in *N. benthamiana* leaves by agroinfiltration as described (Bayless et al. 2016).

### Anti-AAT_Rhg1_ antibody

Affinity-purified anti-AAT_Rhg1_ polyclonal antibodies were raised using the synthetic peptide “Ac-CSKGTPP(dPEG4)C-amide” and validated by multiple methods as described above. For immunoblots, secondary horseradish peroxidase-conjugated goat anti-rabbit was used for detection.

### Transmission electron microscopy

For conventional TEM, soybean root segments in the elongation zone (∼2 mm long) were fixed and stained by standard methods using glutaraldehyde, osmium tetroxide, Epon 812 resin, and uranyl acetate/lead citrate. Representative images were collected from 4 independent root segments for each genotype. Immunodetection TEM experiments were performed similarly to those of Bayless et al. (2019). Soybean (cv. Fayette, Forrest, and Williams 82) root segments previously inoculated with ∼200 J2 SCN (HG 0) per root were hand-sectioned, fixed using glutaraldehyde and paraformaldehyde, embedded in LR White, and sectioned longitudinally with an ultramicrotome. Immunogold labeling used anti-AAT_Rhg1_ and goat anti-rabbit antibody conjugated to 15-nm gold. Anti-AAT_Rhg1_ immunogold particles were counted for single 69 μm^2^ areas within the sampled cells (e.g. cells penetrated by a nematode) and in the identically sized region that had the highest observable signal in directly adjacent cells with normal root cell morphology.

### Immunofluorescent assay

Four days after inoculation SCN-infested root segments were fixed in glutaraldehyde and paraformaldehyde and processed as described in the Supplemental Experimental Procedures. After overnight incubation with anti-AAT_Rhg1_ roots were treated with secondary antibody Alexa Fluor 568 goat anti-rabbit IgG H&L (Abcam ab175471) for 2 h and then imaged right away by confocal microscopy.

### Confocal microscopy

Confocal imaging was performed using a Zeiss inverted laser scanning confocal microscope (ELYRA LSM 780) with a 20× or 40× water immersion objective. For *N. benthamiana* experiments samples were imaged ∼72 h. after agroinfiltration and at least 36 images were assessed for each expression treatment across 3 independent experiments. For FM4-64 imaging, 50 μm FM4-64 solution was infiltrated into transformed leaves 0.5 h before microscopy. For BiFC assays reconstituted YFP signal was acquired using 514 nm laser excitation and a 519 to 620 nm range emission filter and a single detector master gain setting across samples.

### ROS detection in SCN-infested soybean root

About 400 SCN/root were placed near the root tip of whole 2-wk-old soybean seedlings growing in MS media in PlantCon containers and after 3 d the 2 cm root segments with the greatest SCN infestation were harvested. For mock treatments, similar regions of the root were excised. Root segments were incubated with 50 µM H_2_DCFDA (2′,7′-dichlorodihydrofluorescein diacetate, Invitrogen, D399) for 30 min, washed twice for 10 min. and then imaged (Allan and Fluhr 1997; Shin et al. 2005; Chen et al. 2020). Roots were then washed twice for 10 min and imaged using a 10 × water mount objective on the above confocal microscope (488 nm excitation at 2% laser power, 493 to 598 nm emission). At least 16 confocal fluorescent images from 8 different roots across 2 independent replicates per treatment were used for quantification. Percent area of root cells with ROS signal was calculated using ImageJ software as the number of pixels with H_2_DCFDA fluorescent signal intensity above background, compared to the total imaged root area (with SCN bodies and space outside the root tissue excluded).

### MV treatment

MV treatment on *N. benthamiana* leaves was conducted as described (Han et al. 2015). In brief, 20 μm MV solution was infiltrated into the transformed leaves at 64 h post agroinfiltration and followed by 8 h under the previous light conditions to induce internal ROS generation before the confocal analysis.

### Co-immunoprecipitation

Co-IP assays used standard methods (Supplemental Experimental Procedures) on *N. benthamiana* leaf samples taken 60 h after agroinfiltration. Pull-downs utilized prewashed GFP-Trap_A (ChromoTek) beads.

### Compression wounding

*N. benthamiana* leaves were compressed gently for 30 s by full release of a pair of reverse-action tweezers for a consistent wounding force.

### NBT staining

Nitro blue tetrazolium (NBT) staining was performed as described (Han et al. 2015) by incubating buffer-infiltrated leaves in 0.1% w/v NBT for 30 min. followed by fixation and imaging on a flatbed scanner. ImageJ was used to calculate the percentage of dark blue pixels in the agroinfiltrated area. 32 images taken from 12 independent leaves across 3 independent replicates were used for quantification.

## Supplementary Material

kiad098_Supplementary_Data

## Data Availability

All data are summarized either in the main paper or the Supporting Information. Requests for source data should be directed to the corresponding authors.

## Supplemental data

The following materials are available in the online version of this article.

**Supplemental Figure S1**. Confirming the specificity of custom-generated AAT_Rhg1_ antibodies.

**Supplemental Figure S2**. Zoomed-out and zoomed-in images of the same SCN-penetrated cells in WT *rhg1-c* soybean roots, low-copy *rhg1-a*, and high-copy *rhg1-b* soybean roots.

**Supplemental Figure S3**. Little or no immunogold signal in mock-inoculated samples, syncytium cells, or negative controls that omit primary antibody.

**Supplemental Figure S4**. Confirming by competitive binding control that the AATRhg1 antibody is specific in EM antigen detection.

**Supplemental Figure S5**. AATRhg1 signal is not generated by microneedle damage.

**Supplemental Figure S6**. Additional representative TEM micrographs showing large multivesicular compartments common in AATRhg1 overexpression soybean roots (but rarely observed in control roots).

**Supplemental Figure S7**. GFP-AATRhg1 partially co-localizes with ER and PM markers but not with Golgi, plastid, or peroxisome markers in *N. benthamiana* cells.

**Supplemental Figure S8**. Representative confocal micrographs showing that AATRhg1 interaction with GmRBOHG in *N. benthamiana* does not colocalize with a peroxisome marker.

**Supplemental Figure S9**. Additional representative confocal micrographs showing that upon cellular ROS stress, AATRhg1-containing vesicles fuse into larger vesicles through an endocytosis pathway in *N. benthamiana* leaf cells expressing GFP-AAT_Rhg1_ (top set) but not in cells expressing GFP control (second set).
