# Supplementary material for: Soybean transporter AAT Rhg1 abundance increases along the nematode migration path and impacts vesiculation and ROS
Source: Plant Physiol. 2023 Feb 20;192(1):133–53. doi: 10.1093/plphys/kiad098 (PMC10152651; doi:10.1093/plphys/kiad098)
Supplement: kiad098_Supplementary_Data [file kiad098_supplementary_data.pdf]

Supplemental Figures – Han *et al.*
Soybean transporter AAT_{Rhg1} abundance increases along the nematode migration path and impacts vesiculation and ROS

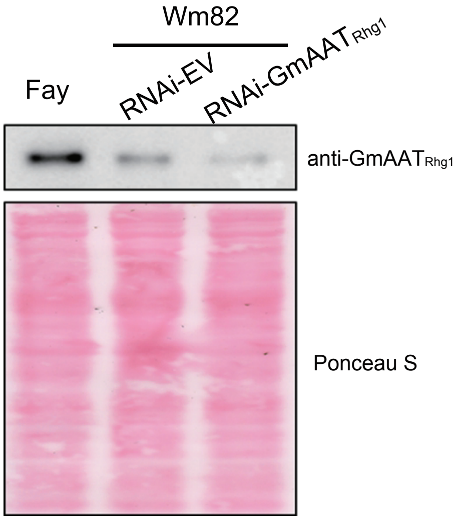

Supplemental Figure S1. Confirming the specificity of custom-generated AAT_{Rhg1} antibodies.
Immunoblot test of anti-AAT_{Rhg1} on root tissue extracts from Fayette (endogenous high-copy *Rhg1*), or transgenic Williams 82 (single-copy *Rhg1*) roots expressing empty silencing control vector (RNAi-EV) or a silencing cassette targeting the AAT_{Rhg1} (RNAi-AAT_{Rhg1}, the same silencing construct used in (Cook et al., 2012)). Immunoblot band migrated at an apparent molecular mass of ~45 kDa relative to SDS-PAGE size markers. Ponceau S staining showed similar total protein loading levels.

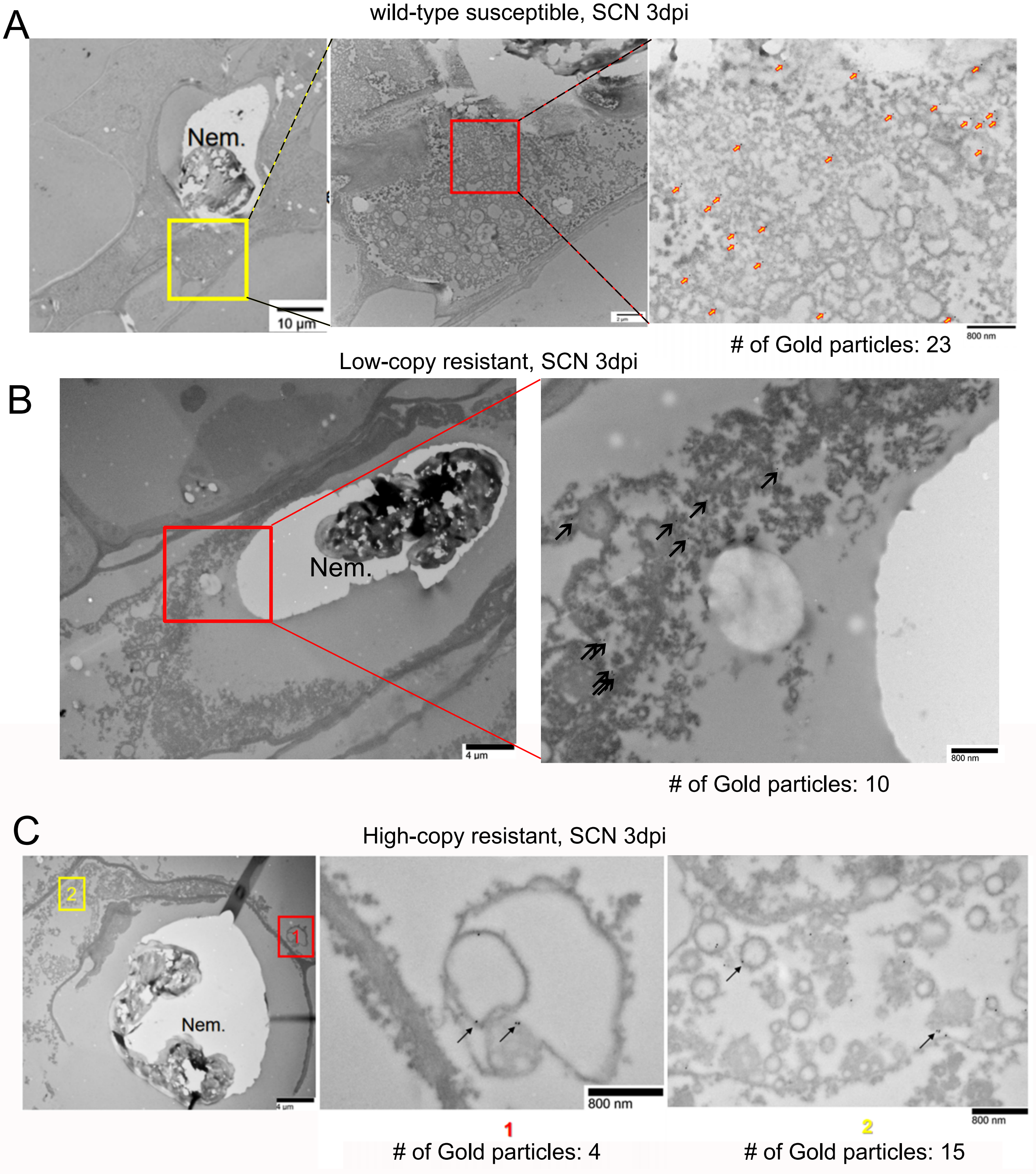

Supplemental Figure S2.
Zoomed-out and zoomed in images of the same SCN-penetrated cells in wild type *rhg1-c* soybean roots (A), low-copy *rhg1-a* (B), and high-copy *rhg1-b* soybean roots (C). Colored boxes show location of zoomed-in figures. Note that A uses same sample as top of Fig. 3A, and image 2 in part C is the same area as shown in Figure 1C. In these experiments, nematode shrinkage during EM sample fixation caused a gap (absence of fixed tissue) in the space occupied by the nematode in live samples.

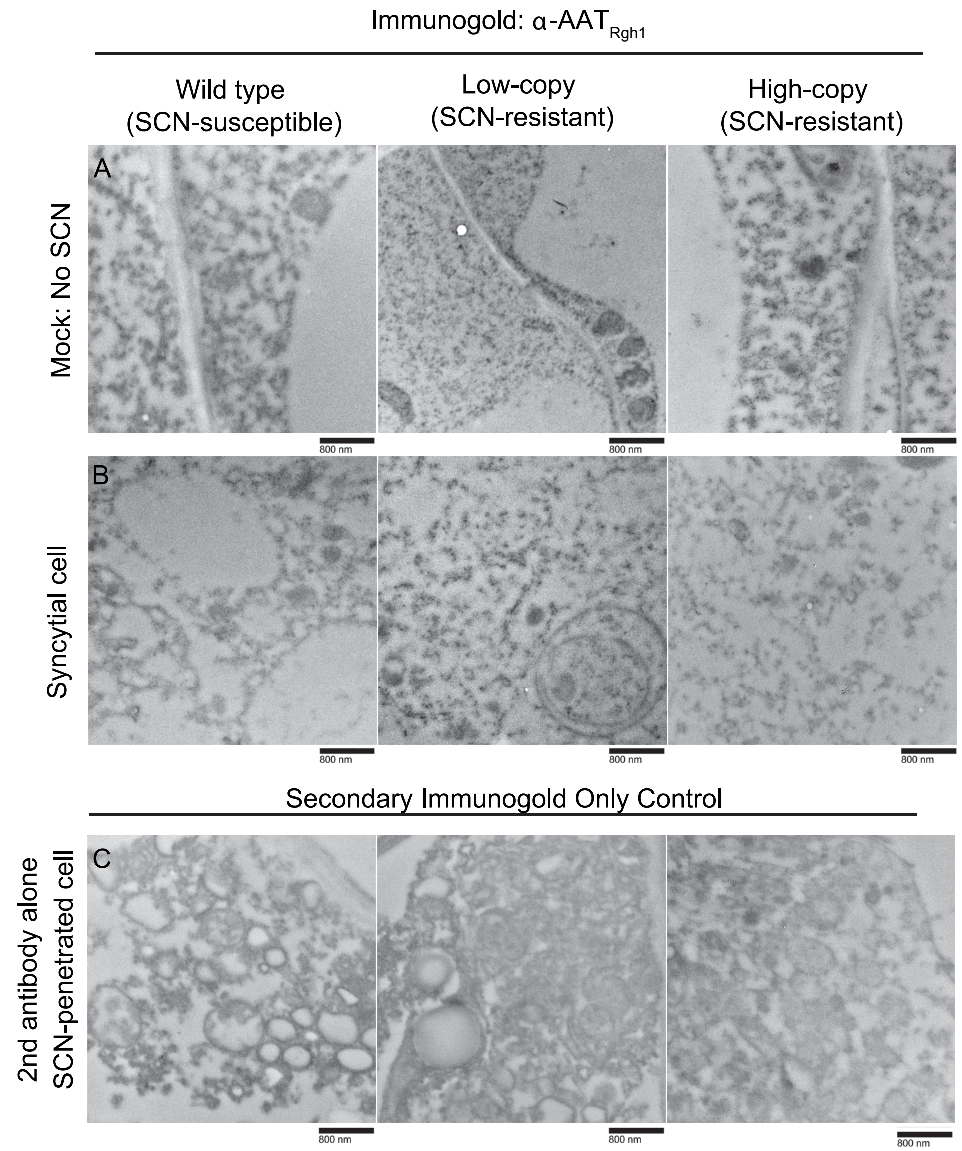

Supplemental Figure S3. Little or no immunogold signal in mock-inoculated samples, syncytium cells, or negative controls that omit primary antibody. Samples were immunolabeled within experiment that produced positive immunogold signal along SCN penetration path as shown in Figure 1.

(A) Electron micrograph of mock-inoculated SCN susceptible (left column), Rhg1 low-copy *Rhg1* (middle column), and high-copy *Rhg1* (right column) roots, with little or no signal observed after immunogold label detection using anti-AAT_{Rhg1} antibody. Scale bar = 800 nm.

(B) Electron micrograph of the syncytium of SCN susceptible (left column), low-copy *Rhg1* (middle column), and high-copy *Rhg1* (right column) roots infested with SCN at 7 dpi, after immunogold label detection using anti-AAT_{Rhg1} antibody. Little to no gold particle labeling was present within syncytia in these or other samples. Scale bar = 800 nm.

(C) Immunogold labeling using only secondary goat anti-rabbit immunodetection antibody only (omitting anti-AAT_{Rhg1} antibody), on root samples taken 7 d after SCN infection: from left to right: SCN susceptible, low-copy *Rhg1* and high-copy *Rhg1* roots. Little or no gold particle labeling was present in SCN-penetrated cells, unlike the frequent labeling observed when anti-AAT_{Rhg1} was included (e.g., Figure 1). Scale bar = 800 nm.

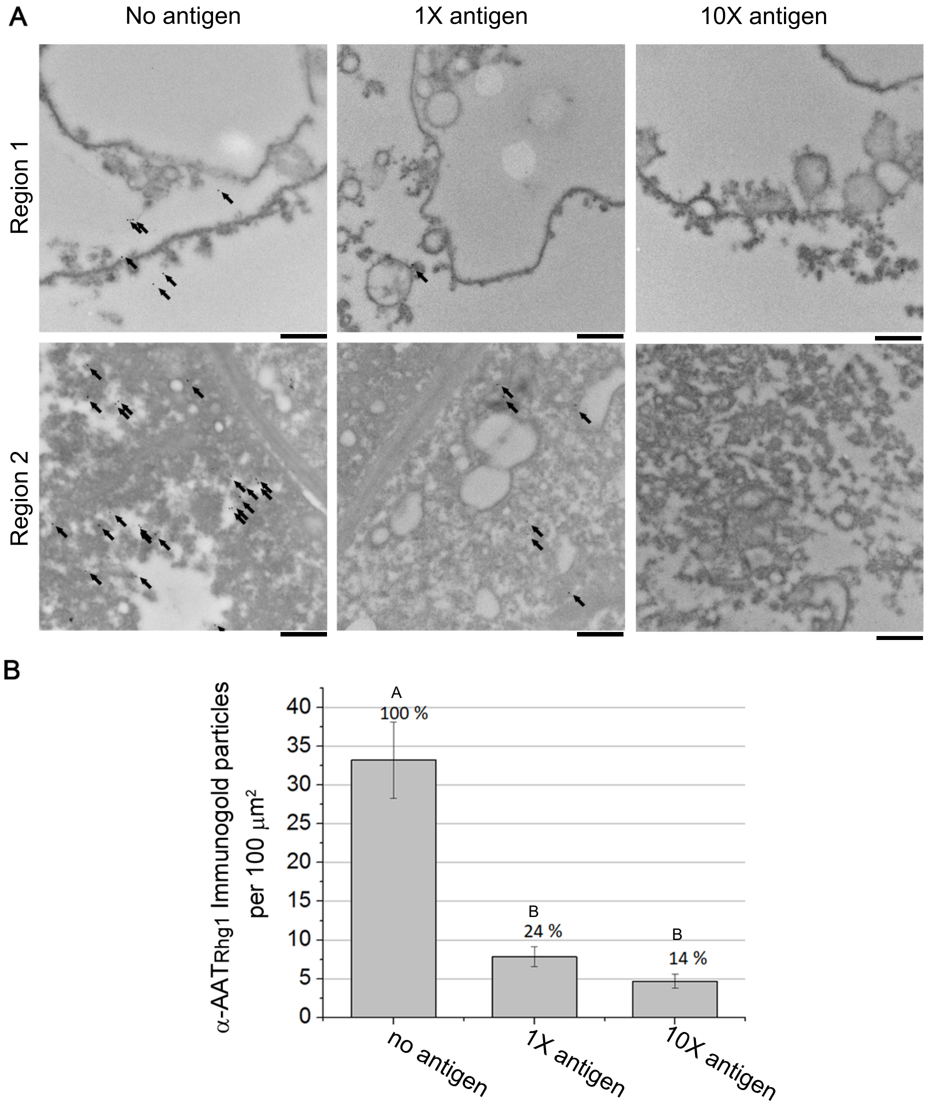

Supplemental Figure 4. Confirming by competitive binding control that the AAT_{Rhg1} antibody is specific in electron microscopy antigen detection.
(A) Representative electron microscopy images (with 15,000X magnification) showing immunogold-labeled AAT_{Rhg1} (left panels) accumulated onto the vesicles membranes of SCN-penetrated cells in SCN infested roots of high-copy *rhg1-b* at 3 dpi. However, only few immunogold-labeled particles could be found on the same region tissue sections mounted onto other separate grids when pre-incubating the anti-AAT_{Rhg1} antibody with 1-fold excess molar antigen (middle panels) or 10-fold excess molar antigen (right panels). Arrows indicate immunogold particles in each images. Scale bars = 800 nm.
(B) Numbers of AAT_{Rhg1} immunogold particles detected in a similar 100 μm^2 area were compared between grids of the same region tissue sections with no antigen, 1X antigen or 10x antigen competitive binding treatment. At least 10 different images, from two independent experiments, were used to count AAT_{Rhg1} immunogold particles per treatment. Values are mean ± SE. Treatments marked with the same letter were not significantly different (ANOVA, $P < 0.01$).

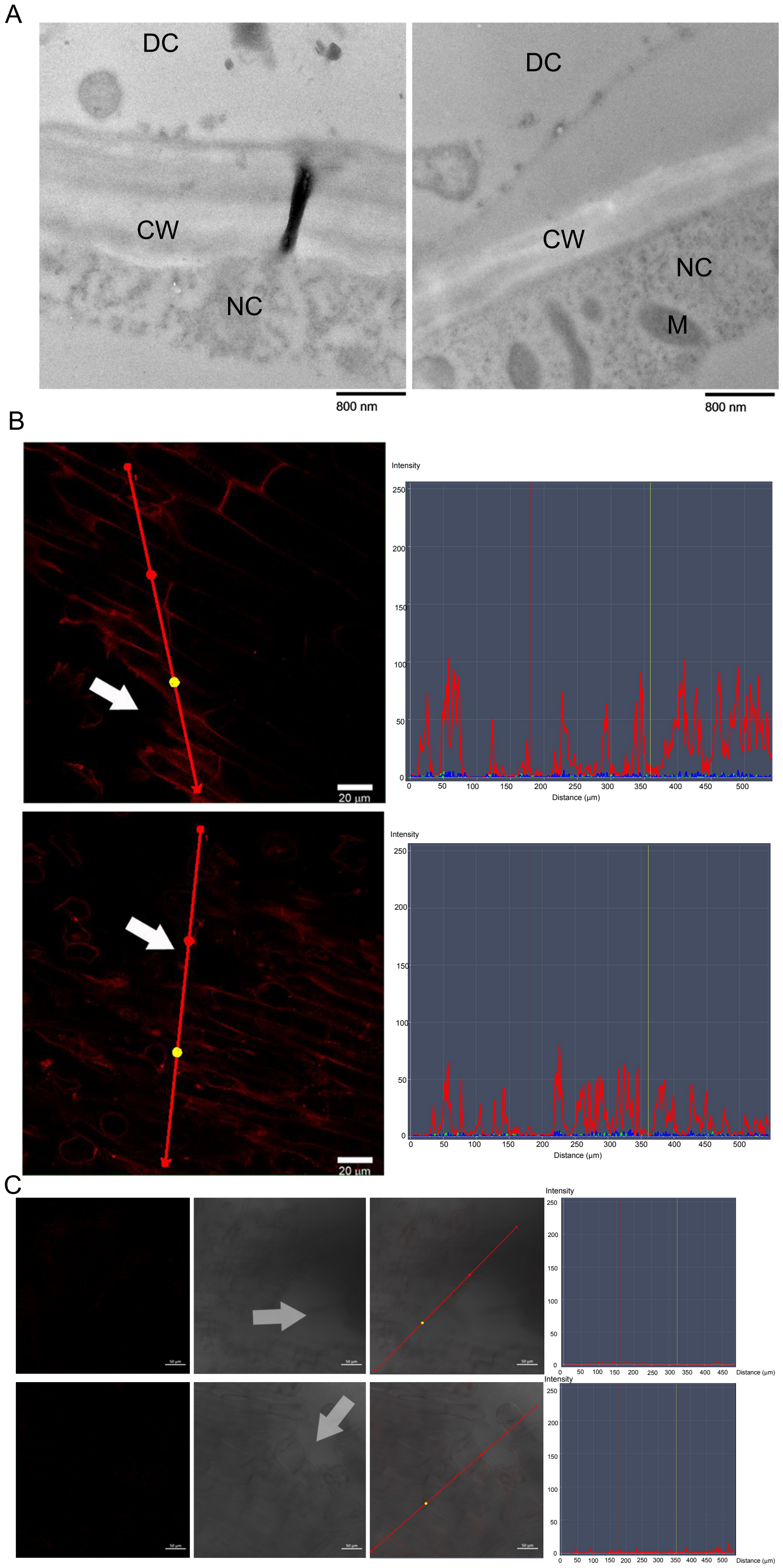

Supplemental Figure S5. AAT_{Rhg1} signal is not generated by microneedle damage.

(A) Representative electron micrograph showing that only rare immunolabeled anti-AAT_{Rhg1} gold particles could be found in microneedle damaged cells (DC) as well as in normal cells (NC) in high-copy *Rhg1* soybean roots. DC, microneedle damaged cell; NC, normal cell; CW, cell wall; M, mitochondrion. Scale bars = 800 nm.

(B) Representative immunofluorescent images indicated no specific anti-AAT_{Rhg1} fluorescent signal could be observed in microneedle damaged cells. Higher magnification used to highlight the wounded area. White arrow: gap caused by microneedle. Dots on red line mark location for lines on adjacent graph. Note that a 40x objective was used and the fluorescence detection sensitity was set higher in these experiments than in those of Figure 2 so that weak background fluorescence was visible; no elevated fluorescence signal was observed at the sites of needle damage. Scale bars = 20 µm.

(C) Additional immunofluorescent images using the same magnification (under a 20x objective) as Figure 2 indicate no specific anti-AAT_{Rhg1} fluorescent signal could be observed in microneedle damaged cells. Scale bars = 50 µm.

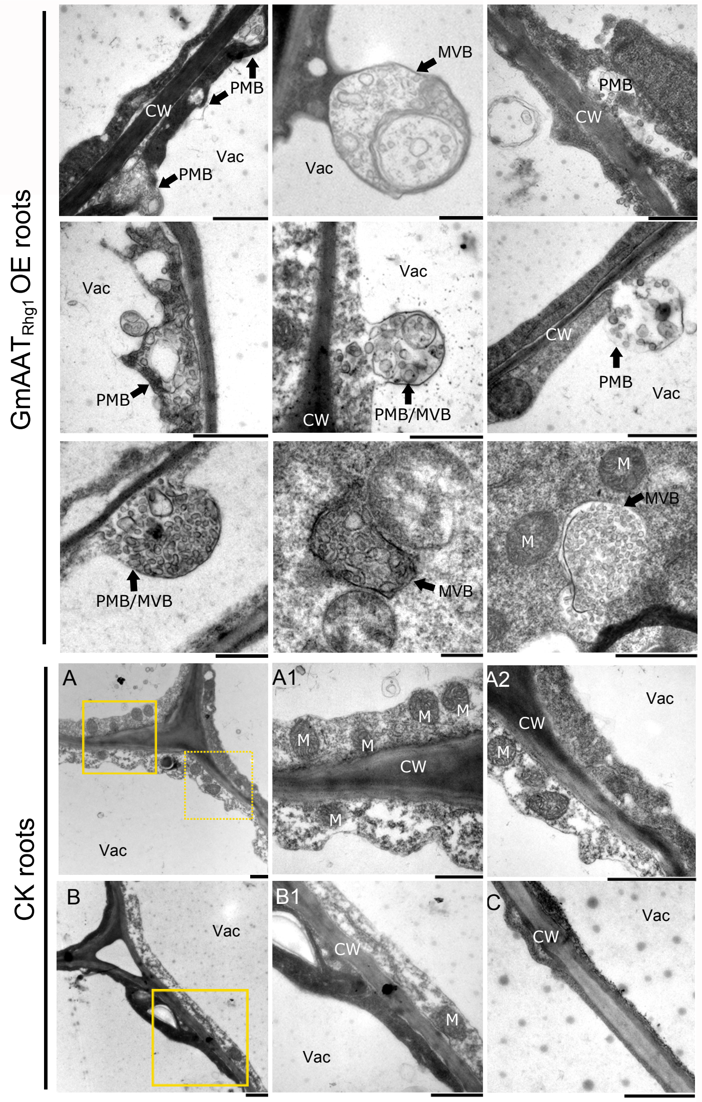

Supplemental Figure S6. Additional representative TEM micrographs showing large multivesicular compartments common in AAT$_{Rhg1}$ overexpression soybean roots (but rarely observed in control roots).
Top three rows: Representative TEM images from AAT$_{Rhg1}$ transformed root cells. Different types of VLBs located inside the cytoplasm, and PMB observed outside of cell and in transitional MVB fused with cell membrane.
Bottom Panel: Representative TEM images of transgenic soybean root cells (elongation region) with control vector overexpression. (A) three neighboring cells with cytoplasm and vacuole. Framed zones were further enlarged and shown in A1 and A2, solid line frame and dash line frame, respectively. (B and C) cellular contents in transformed control roots. (B1) enlarged zone of the frame area in B.
CW, cell wall; M, mitochondrion; MVB, multivesicular body; PMB, paramural body; Vac, vacuole. Scale bars = 1 μm.

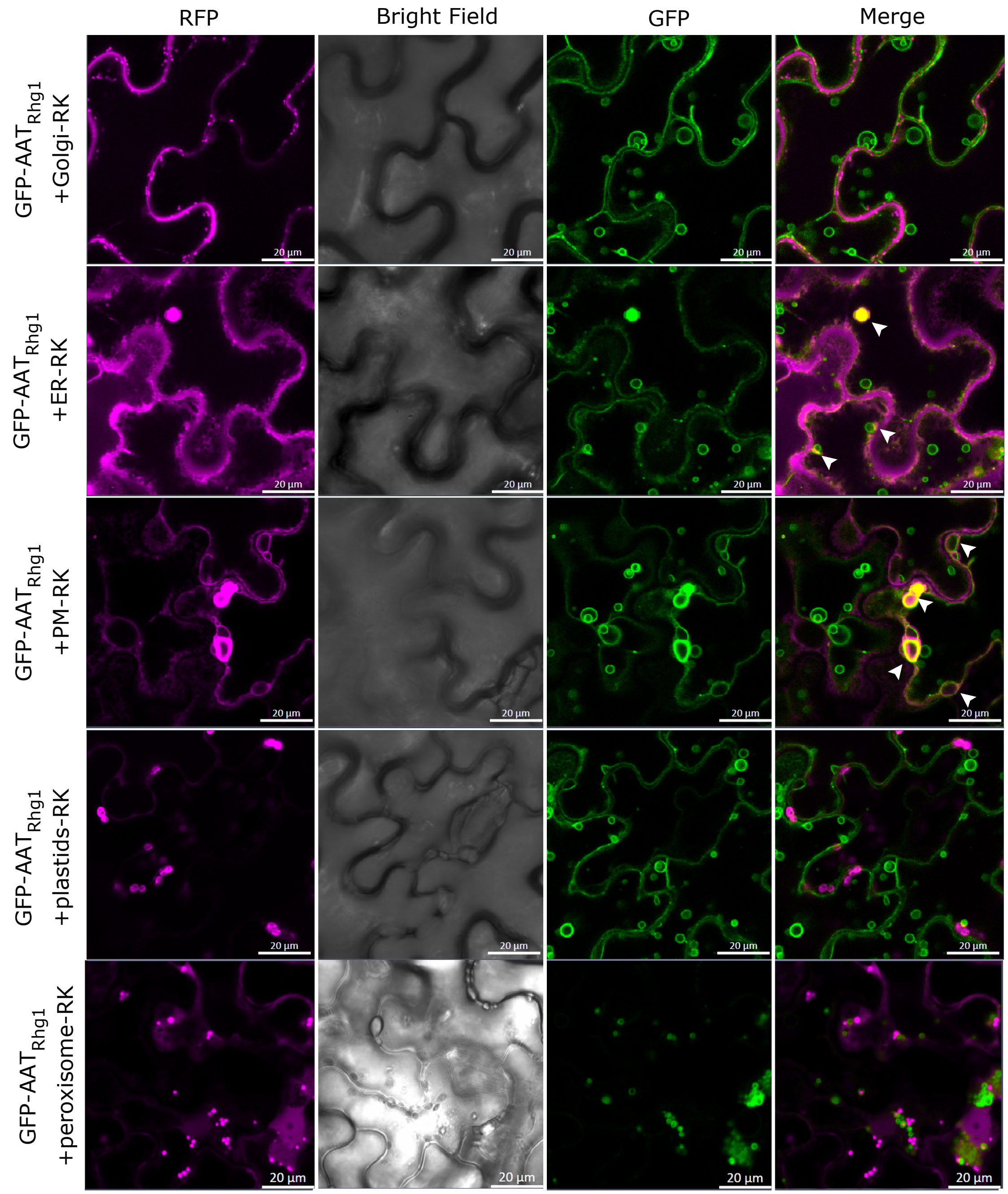

Supplemental Figure S7. GFP-AAT$_{Rhg1}$ partially co-localizes with ER and PM markers but not with Golgi, plastid or peroxisome markers in *N. benthamiana* cells.
Live imaging of agroinfiltrated *N. benthamiana* leaves transiently expressing different combinations of protein fusions of green fluorescent protein tagged AAT$_{Rhg1}$, red fluorescent protein tagged Golgi marker (Golgi-RK), ER marker (ER-RK), plasma membrane marker (PM-RK), plastids marker (plastids -RK) or peroxisome marker (peroxisome-RK). The emission channel for RFP is shown in magenta, the bright field channel in gray, and the GFP channel in green. Imaging was performed at 2 days post-infiltration. Representative images from leaves of four individual plants, in three independent experiments. Scale bars = 20 µm.

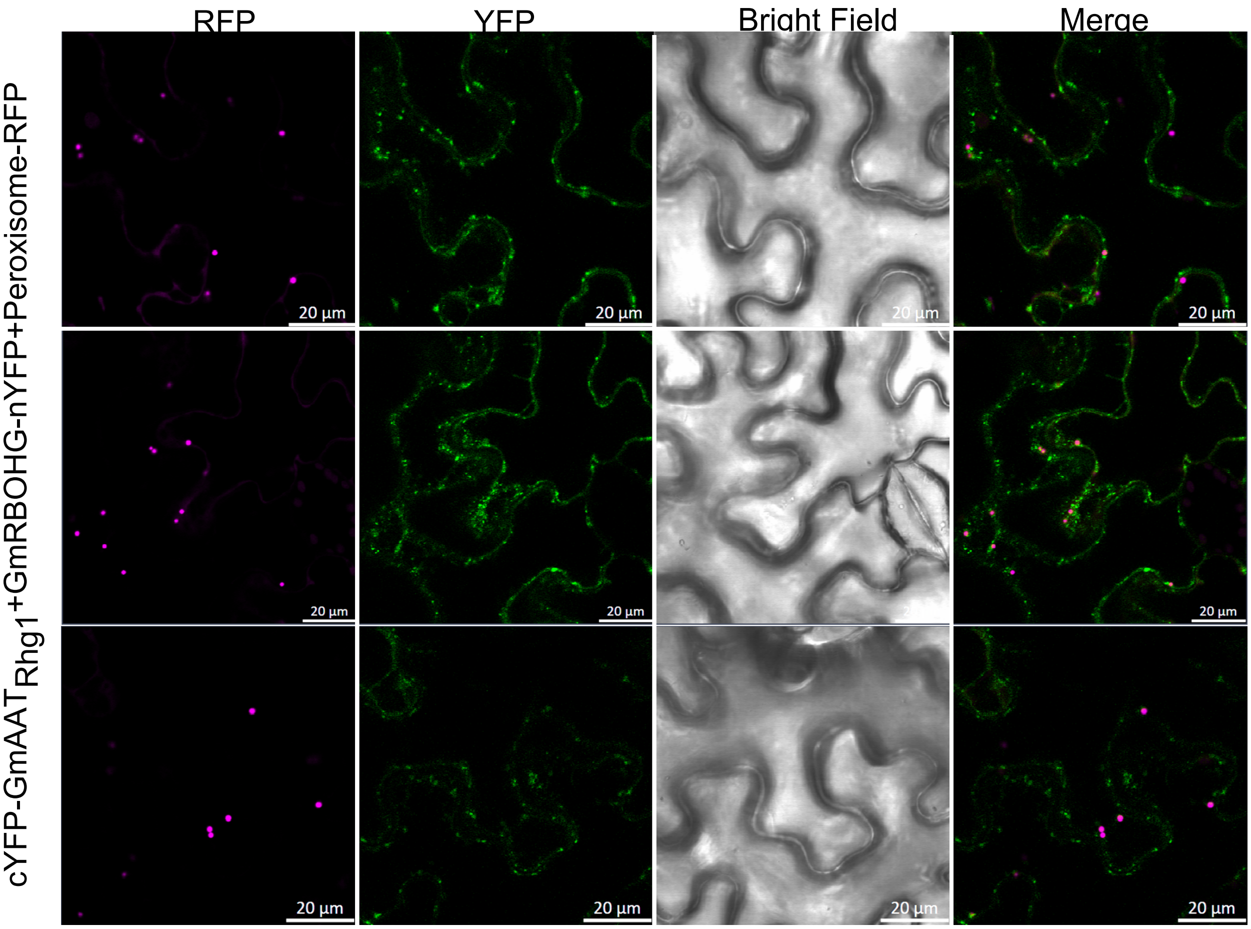

Supplemental Figure S8. Representative confocal micrographs showing that AAT$_{Rhg1}$ interaction with GmRBOHG in *N. benthamiana* does not colocalize with a peroxisome marker. In cells coexpressing cYFP-AATRhg1 and GmRBOHG-nYFP, complemented YFP fluorescence signal was detected in small vesicles (second column, green-labeled), and the peroxisomes were labeled in magenta (first column). In the merged image, overlapping location of green and magenta signal would be colored white. The experiments were repeated on three separate dates with similar results. Scale bars = 20 µm.

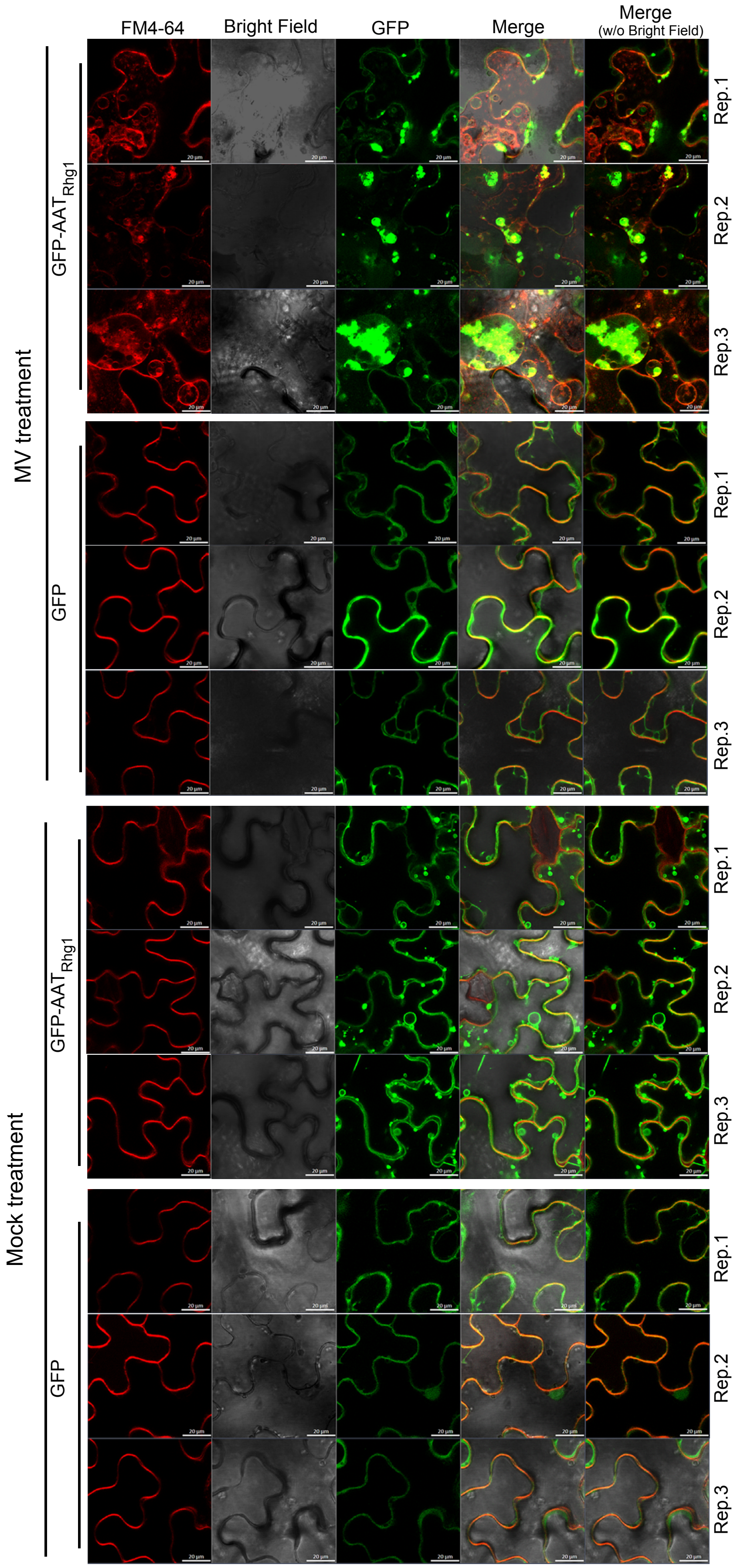

Supplemental Figure S9. Additional representative confocal micrographs showing that upon cellular ROS stress, AAT_{Rhg1}-containing vesicles fuse into larger vesicles through an endocytosis pathway in *N. benthamiana* leaf cells expressing GFP-AAT_{Rhg1} (top set) but not in cells expressing GFP control (second set). Third and fourth set show leaf cells expressing GFP-AAT_{Rhg1} or GFP, with no added ROS stress. MV treatment: methyl viologen (inducer of superoxide and other ROS). Scale bars = 20 µm.

Supplemental Methods S1

Han *et al.* "Soybean transporter AAT_{Rhg1} abundance increases along the nematode migration path and impacts vesiculation and ROS"

Nematode inoculum

SCN eggs of Hg 0 populations were obtained from Alison Colgrove at the University of Illinois Plant Clinic. Eggs were incubated in hatching buffer (3 mM $ZnCl_2$) for 5 days at room temperature. Infectious J2 SCN were obtained and surface-disinfested with sterilization buffer (0.1g/L $HgCl_2$ and 0.01% (w/v) Sodium Azide) for three minutes. After rinsing twice in water, J2 SCN were resuspended in 0.05% (w/v) sterile agarose water for root inoculation.

Plasmid constructs.

For transient overexpression vectors, the soybean AAT_{Rhg1} and GmRBOHG (*Glyma.06G162300.1*) ORFs were PCR-amplified from Williams82 cDNA generated by the iScript cDNA Synthesis Kit(Bio-Rad) and KAPA HiFi polymerase (Kapa Biosystems). Transient overexpression of soybean AAT_{Rhg1} and GmRBOHG was performed by assembling each respective ORF with the double CaMV 35S promoter with TMV omega enhancer (pICH51288) and nopaline synthase (NOS) terminator into the binary vector pAGM4673 (MoClo Tool Kit) using the Golden Gate cloning method (Weber et al., 2011).

For BiFC vectors, nYFP or cYFP fusion expression constructs driven by CaMV 35S promoter were prepared similarly as (Zhao et al., 2013). A pCAMBIA2300 base vector was used. ORFs encoding AAT_{Rhg1} or α-$SNAP_{Rhg1}$WT with stop codon, or GmRBOHG or NSF without stop codon were flanked by specific LIC1 adaptor at 5'(5'-C gAC gAC AAg ACC gTg ACC-3') and LIC2 adaptor at 3'(5'-gA ggA gAA gAg CCg Tcg-3') by overhang PCR amplification and purified by gel-extraction using QIAquick Gel Extraction Kit (Qiagen). Then, a Ligase Independent Cloning (LIC) method was performed to fuse cYFP to the N-terminus of AAT_{Rhg1} or α-$SNAP_{Rhg1WT}$, and to fuse nYFP to the C-terminus of NSF or GmRBOHG, as described (Xu et al., 2010).

For co-IP vectors, a construct encoding N-terminal GFP translationally fused to full length AAT_{Rhg1} (using an AAT_{Rhg1} cDNA with stop codon) was cloned into a binary expression construct pJG045, driven by a CaMV 35S promoter, using LIC (ligation-independent cloning) methods (Du et al., 2013). Similarly, a construct encoding N-terminal GmRBOHG without stop codon fused to C-terminal 6X MYC tag was cloned into pJG045 using LIC methods.

N. benthamiana experiments

N. benthamiana plants with 2-3 fully expanded leaves were used for agroinfiltration with *Agrobacterium tumefaciens* strain GV3101(pMP90) as described in (Bayless et al., 2016). The subcellular compartment marker protein constructs expressed first 49 AA of GmMan1 (soybean α-1,2-mannosidase) as Golgi marker, chimeric signal peptide of AtWAK2 at the N-termnus of the RFP and the ER retention signal HDEL at the C-terminus as the ER marker, full length AtPIP2A as PM marker, and first 79 AA of small subunit of tobacco rubisco as plastid marker (Nelson et al., 2007)

Antibody Production

Affinity-purified polyclonal antibodies, raised in rabbit against the synthetic peptide "SKGTPP" matching residues 15-20 near the N-terminus of $GmAAT_{Rhg1}$, were produced by New England Peptide. Antibody specificity was validated using immunoblots with root lysates of ten-copy *Rhg1* Fayette) compared to single-copy *Rhg1* Williams 82 roots, and to Williams 82 roots expressing an RNAi gene silencing cassette targeting endogenous AAT_{Rhg1} (Figure S1).

Immunoblots with anti-AAT_{Rhg1}

Soybean root samples were frozen in liquid nitrogen and extracted in buffer containing 50 mM Tris·HCl (pH 7.5), 150 mM NaCl, 5 mM EDTA, 0.2% (v/v) Triton X-100, 10% (v/v) glycerol, and protease inhibitor mixture (Sigma, P9599). Protein was extracted by homogenization in a PowerLyzer 24 (MO BIO) at 2000 rpm for three cycles with 15 s interval. Each sample was quantified by Bradford assays to achieve equal loading of total protein on SDS/PAGE gels. Blots were incubated with anti-AAT_{Rhg1} antibody in 5% (w/v) nonfat dry milk TBS-T (50 mM Tris, 150 mM NaCl, 0.05% (v/v) Tween 20) at 1:1,000 overnight at 4°C. After four washes with TBS-T,

secondary horseradish peroxidase-conjugated goat anti-rabbit was added at 1:10,000 and incubated for 1h at room temperature with mild agitation on a horizontal shaker. Blots were then washed with TBS-T four times, followed by chemiluminescence detection with SuperSignal West Pico or Dura chemiluminescent substrate (Thermo Scientific). Blots were imaged using a ChemiDoc MP chemiluminescent imager (Bio-Rad).

Generation of transgenic soybean roots

As previously described, transgenic roots were generated with *Agrobacterium rhizogenes* strain ArQua1(Melito et al., 2010). The same overexpression level 1 transcription unit used to overexpress AAT_{Rhg1} in *N. benthamiana* described above was cloned with a 35S::GFP marker for transgenic root identification into pAGM4673 by Golden Gate assembly. Cotyledons from Wm82 soybeans were transformed with either GFP empty vector or GFP/AAT_{Rhg1} overexpression vector to generate roots as previously described (Melito *et al.*, 2010).

Conventional TEM

GFP-positive soybean root samples were collected 14 days after transformation. Root segments in the elongation zone about 2 mm long were vacuum infiltrated with 2.5% glutaraldehyde. After being stained with 1% (w/v) osmium tetroxide for 4 hrs., the samples were rinsed with 100 mM phosphate buffer (pH 7.0) five times with a 30-min interval. The samples were dehydrated in a graded ethanol series (50%, 70%, 80%, 90%, 95%, and 100% v/v) and pure acetone for 40 min. Then the samples were embedded with Epon 812 resin (SPI supplies Inc. PA, USA). Ultra-thin sections (100 nm thickness) were cut using a Leica UC 6 microtome and placed onto 100 mesh carbon-covered copper grids. Before the examination, grids were stained with 2% uranyl acetate and 2% (w/v) lead citrate for 15 min each. An H-7650 TEM (Hitachi, Ibaraki, Japan) at 80 KV equipped with a Gatan 830 CCD camera (Gatan, USA) was used for examination and photography. Representative images were collected from four independent root segments for each genotype.

Electron Microscopy and Immunolabeling.

Immunolabeling were performed similarly to (Bayless et al., 2019). Segments from roots (Fayette, Forrest and Williams 82) previously inoculated with ~200 J2 SCN (Hg 0) per root were hand-sectioned with a razor at the indicated dpi. Root sections about 2 mm long were vacuum infiltrated in fixation buffer (0.1% (v/v) glutaraldehyde and 4% (v/v) paraformaldehyde in 0.1M sodium phosphate buffer (PB) pH 7.4) and incubated overnight. After dehydration in 50%, 70%, 90%, 95% and 100% ethanol series, samples were embedded in LR White. Ultrathin sections (~90-nm) were taken longitudinally with an ultramicrotome (UC-6; Leica). For the immunogold labeling, samples were mounted on nickel slot grids. Grids were first activated on drops of 50 mM glycine/PBS for 15 min, and then blocked in drops of blocking solutions for goat gold conjugates (Aurion) for 30 min and then equilibrated in 0.1% (w/v) BSA-C/PBS (incubation buffer). Next, grids were incubated with the anti-AAT_{Rhg1} antibodies diluted 1:1000 (in incubation buffer) overnight at 4 °C. After washing five times in incubation buffer, grids were incubated for 2 h with goat anti-rabbit antibody conjugated to 15-nm gold (Aurion) diluted 1:50 in incubation buffer. After six washes in incubation buffer and two washes in PBS, grids were fixed using 2.0% (v/v) glutaraldehyde in 0.1 M phosphate buffer for 5 min. Finally, grids were further washed twice in 0.1 M phosphate buffer for five minutes each and then five 2-minute washes in water. Images were collected with a MegaView III digital camera on a Philips CM120 transmission electron microscope. Anti-AAT_{Rhg1} immunogold particles were counted for single 69 μm^2 areas within the sampled cells (e.g., cells penetrated by a nematode) and in the identically-sized region that had the highest observable signal in directly adjacent cells with normal root cell morphology (large central vacuole).

Immunofluorescent assay

4 dpi SCN-infested roots segments were fixed in 0.1% (v/v) glutaraldehyde and 4% (v/v) paraformaldehyde in 0.1M sodium phosphate buffer (PB) (pH 7.4) overnight after vacuum infiltration for about 1 hour as described above. For immunofluorescence processing, the fixed roots segments were briefly rinsed with PBT buffer (1XPBS pH=7.4, 1% (w/v) BSA and 0.1% (v/v) Triton-X100) and then blocked with PBT blocking solutions (PBS pH=7.4, 1% (w/v) BSA and 0.1% Triton-X100, plus 5 % goat serum (Sigma-Aldrich) overnight at 4 °C. The root segments were incubated with the primary antibody diluted 1:1000 in PBT blocking solution at 4 °C overnight.

Next, the incubated roots were washed 5 times for 10 min. each with PBT buffer at 800 rpm on a shaker platform at room temperature. Roots were then incubated with 0.4 µg/ml secondary antibody Alexa Fluor 568 goat anti-rabbit IgG H&L (Abcam ab175471) in PBT at for 2 hrs at room temperature, covered with a foil to shield the 2nd antibody solutions from light. After again washing with PBT for 10 mins 5 times at room temperature, the samples could be imaged by confocal microscopy right away.

Confocal Microscopy

Confocal imaging was performed using an inverted Carl Zeiss laser-scanning confocal microscope (ELYRA LSM 780) with a 20× or 40× water immersion objective. All *A. tumefaciens*-transformed leaves were excised using a paper punch and monitored at ~72 hr after infiltration. For green fluorescent protein detection, GFP or GFP tagged chimera protein was excited at the wavelength of 488 nm, and the emitted fluorescence was detected with a 493-594 nm emission filter. Chloroplast autofluorescence was excited at 405 nm and detected at 635 to 708 nm to determine the position of chloroplasts for reference. For FM4-64 imaging, 50 µm FM4-64 solution (Invitrogen/Molecular Probes; T13320) was inoculated additionally into transformed leaves 0.5 hr before observation under the confocal laser scanning microscope. FM4-64 stained leaf tissues were excited with an excitation laser of 514 nm, and the emission signals were collected at 592-651 nm. For immunofluorescence, the Alexa Fluor 568 immunodetected plant tissue was excited at the wavelength of 561 nm and detection wavelength was at the range of 568-640 nm. For BiFC assay, YFP recombinant signal was acquired using a 514 nm laser for excitation combined with a 519-620 nm range emission filter. Images were collected using a standardized scan area of 442.2 × 442.2 µm (for 20× objective used) or 212.55 ×212.55 µm (40× water immersion objective), with a 1024 × 1024 pixels size frame. The detector master gain setting was from 700 to 800 dependent on different fluorescent signal intensity, and 1.01 AU size of pinhole was used for all the *Nicotiana benthamiana* samples, and 2.36 AU was used for soybean root immunofluorescence assays. At least 36 images were assessed for each expression treatment across three independent experiments.

H_2DCFDA detection of ROS in SCN-infested soybean roots

Whole 2-week old soybean seedlings germinated in PlantCon containers (MP bio, Cat#2672202) were used for SCN inoculation or mock treatment. About 400 SCN/root were placed near the vicinity of each root tip by pipette at day 0. After three days, the 2 cm root segments with greatest SCN infestation were harvested. For mock treatments, similar regions of the root were excised. Detached roots were then incubated in 1X PBS (Phosphate Buffered Saline) buffer with 50 μM H_2DCFDA (2’, 7’-dichlorodihydrofluorescein diacetate, Invitrogen, D399) for 30 min shaking at 200 rpm/min at room temperature (Allan and Fluhr, 1997; Chen et al., 2020; Shin et al., 2005). Roots were then washed twice with 1X PBS, 10 min each time, and imaged. A Zeiss LSM 780 confocal microscope (ELYRA) was used with a 10× objective. H_2DCFDA was excited at 488 nm at 2% laser power and 493-598 nm emission was detected. At least 16 confocal fluorescent images from 8 different roots across two independent replicates per treatment were used for quantification. The area with H_2DCFDA fluorescent was calculated using ImageJ software as the number of pixels with signal intensity above background, compared to the total imaged root area (with SCN bodies and space outside the root tissue excluded) in order to calculate the percent area of root cells with ROS signals.

MV treatment

Methyl Viologen (MV) treatment on *N. benthamiana* leaves was conducted as described (Han et al., 2015). In brief, 20 μm MV solution was infiltrated into the transformed leaves at 64 hr post agroinfiltration and followed by 8 hr under the previous light conditions to induce internal ROS generation before the confocal analysis.

Coimmunoprecipitation

For co-IP assays, 4-wk-old fully expanded *N. benthamiana* leaves were used for agroinoculation at OD 0.6 for total 60-hr expression. About 2 g *N. benthamiana* leaf tissues for each treatment was collected from four different plants as one biological replicate. After chilling by liquid nitrogen, the tissue was ground by hand using a pestle in a pre-chilled mortar. Then 4 ml of protein extraction buffer (50 mM Tris·HCl (pH

7.5), 150 mM NaCl, 5 mM EDTA, 0.2% (v/v) Triton X-100, 10% (v/v) glycerol, 1/100 Sigma protease inhibitor cocktail) was added to the mortar and the sample was further homogenized by grinding. The lysates were transferred into tubes and spun down at 6000 G for 10 min at 4°C three times to remove insoluble debris. The resulting supernatant was incubated with prewashed GFP-Trap_A (ChromoTek) beads for 3 h at 4°C. The precipitations were washed four times with ice-cold immunoprecipitation buffer at 4°C and were analyzed by immunoblot using anti-Myc (Sigma), or anti-GFP (Cell Signaling Technology) antibodies. Secondary horseradish peroxidase-conjugated goat anti-rabbit IgG (Sigma) was used to detect the primary anti-AAT_{Rhg1} antibody derived from rabbit. Chemiluminescence detection was performed with SuperSignal Dura chemiluminescent substrate (Thermo Scientific) and developed by a ChemiDoc MP chemiluminescent imager (Bio-Rad).

Wounding treatment

For wounding, each *N. benthamiana* leaves was compressed gently for 30 seconds using reverse-action tweezers. By the full release of the reverse-action tweezers, a consistent wounding force was provided across all the samples.

NBT staining

Nitro blue tetrazolium (NBT) staining was performed as described (Han *et al.*, 2015). In brief, *N. benthamiana* leaves were detached and vacuum-infiltrated with 10 mM NaN_3 in 10 mM potassium phosphate buffer (8.6 mM K_2HPO_4 and 1.4 mM KH_2PO_4) pH 7.8, for 1 min. Then, the fully infiltrated leaves were transferred into 0.1% (w/v) NBT (in 10 mM potassium phosphate buffer pH 7.8) and put on a platform shaker shaking at 150 rpm for 30 min at room temperature, under a foil cover to reduce light exposure. The stained leaves were then cleared by boiling in destaining buffer (acetic acid: glycerol: ethanol (1:1:3 [v/v/v]). Photographs were obtained by scanning with a flatbed scanner (EPSON, V500 PHOTO SCANNER) at 800 dpi and NBT stain measurements were obtained using ImageJ (https://imagej.nih.gov/ij/). The region for evaluation was matched to the outline of the full agroinfiltrated area, and pixel intensities were obtained for each individual image. Number of dark blue-stained pixels in an image was divided by total analyzed pixels (the total infiltration area) to calculate the percent

area with NBT stain. 32 images taken from 12 independent leaves across three independent replicates were used for quantification.

References for Supplemental Experimental Procedures

Allan, A.C., and Fluhr, R. (1997). Two distinct sources of elicited reactive oxygen species in tobacco epidermal cells. The Plant Cell **9**:1559-1572.

Bayless, A.M., Zapotocny, R.W., Han, S., Grunwald, D.J., Amundson, K.K., and Bent, A.F. (2019). The rhg1-a (Rhg1 low-copy) nematode resistance source harbors a copia-family retrotransposon within the Rhg1-encoded α-SNAP gene. Plant Direct **3**:e00164.

Bayless, A.M., Smith, J.M., Song, J., McMinn, P.H., Teillet, A., August, B.K., and Bent, A.F. (2016). Disease resistance through impairment of α-SNAP–NSF interaction and vesicular trafficking by soybean Rhg1. Proceedings of the National Academy of Sciences **113**:E7375-E7382.

Chen, X., Li, S., Zhao, X., Zhu, X., Wang, Y., Xuan, Y., Liu, X., Fan, H., Chen, L., and Duan, Y. (2020). Modulation of (Homo) Glutathione Metabolism and H_2O_2 Accumulation during Soybean Cyst Nematode Infections in Susceptible and Resistant Soybean Cultivars. International Journal of Molecular Sciences **21**:388.

Du, Y., Zhao, J., Chen, T., Liu, Q., Zhang, H., Wang, Y., Hong, Y., Xiao, F., Zhang, L., Shen, Q., et al. (2013). Type I J-domain NbMIP1 proteins are required for both Tobacco mosaic virus infection and plant innate immunity. PLoS Pathogens **9**.

Han, S., Wang, Y., Zheng, X., Jia, Q., Zhao, J., Bai, F., Hong, Y., and Liu, Y. (2015). Cytoplastic glyceraldehyde-3-phosphate dehydrogenases interact with ATG3 to negatively regulate autophagy and immunity in Nicotiana benthamiana. The Plant Cell **27**:1316-1331.

Melito, S., Heuberger, A.L., Cook, D., Diers, B.W., MacGuidwin, A.E., and Bent, A.F. (2010). A nematode demographics assay in transgenic roots reveals no significant impacts of the Rhg1 locus LRR-Kinase on soybean cyst nematode resistance. BMC plant biology **10**:1-14.

Nelson, B., Cai, X., and Nebenführ, A. (2007). A multicolored set of in vivo organelle markers for co-localization studies in Arabidopsis and other plants. The Plant Journal **51**:1126-1136.

Shin, R., Berg, R.H., and Schachtman, D.P. (2005). Reactive oxygen species and root hairs in Arabidopsis root response to nitrogen, phosphorus and potassium deficiency. Plant and Cell Physiology **46**:1350-1357.

Weber, E., Engler, C., Gruetzner, R., Werner, S., and Marillonnet, S. (2011). A modular cloning system for standardized assembly of multigene constructs. PloS One **6**.

Xu, G., Sui, N., Tang, Y., Xie, K., Lai, Y., and Liu, Y. (2010). One-step, zero-background ligation-independent cloning intron-containing hairpin RNA constructs for RNAi in plants. New Phytologist **187**:240-250.

Zhao, J., Liu, Q., Zhang, H., Jia, Q., Hong, Y., and Liu, Y. (2013). The rubisco small subunit is involved in tobamovirus movement and Tm-22-mediated extreme resistance. Plant Physiology **161**:374-383.

Oligonucleotide primer name	Sequence
lic1+ GmAAT forward	C gAC gAC AAg ACC gT g ACC ATG TCT CCG GCC GCC GG
lic2+ GmAAT reverse without stop codon	gA ggA gAA gAg CCg TGA CTT GCT ACT AAA AGCATTATATATG
lic2+ GmAAT reverse with stop codon	gA ggA gAA gAg CCg TTA TGA CTT GCT ACT AAA AGCATTATATATG
cdna rbohG forward	ATGGTAACAGAAGAGGTTGTTGGAGGA
cdna rbohG reverse	TTAGAAATTTTCTTTGTGGAAATCATATTT
lic1+ Glyma.06G162300 GmRbohG forward	C gAC gAC AAg ACC gT g ACC ATG GTA ACA GAA GAG GTT
lic2+ Glyma.06G162300 CDS GmRbohC2 reverse without stop codon	gA ggA gAA gAg CCg TTG GAA ATT TTC TTT GTG GAA AT
lic1+ alpha-SNAP wt forward	C gAC gAC AAg ACC gT g ACC ATGGCCGATCAGTTATCGAA
lic2+ alpha-SNAP wt reverse with stop codon	TCAAGTAAGATCATCCTCCTCAAGTTCT
lic1+ NSF forward	C gAC gAC AAg ACC gT g ACC ATGGCGAGTCGGTTCGGGTTATCGTCT
lic2+ NSF reverse without stop codon	gA ggA gAA gAg CCg TAA CCT AACAACATCCTGGAGGCAATCATAGAAATGAGCG
